# Tri-Planar Geometric Dimensioning and Tolerancing Characteristics of SS 316L Laser Powder Bed Fusion Process Test Artifacts and Effect of Base Plate Removal

**DOI:** 10.3390/ma14133575

**Published:** 2021-06-26

**Authors:** Baltej Singh Rupal, Tegbir Singh, Tonya Wolfe, Marc Secanell, Ahmed Jawad Qureshi

**Affiliations:** 1 Additive Design and Manufacturing Systems (ADaMS) Lab, Department of Mechanical Engineering, University of Alberta, Edmonton, AB T6G 2G8, Canada; baltej@ualberta.ca; 2 Energy Systems Design Lab (ESDL), Department of Mechanical Engineering, University of Alberta, Edmonton, AB T6G 2G8, Canada; secanell@ualberta.ca; 3 InnoTech Alberta, Surface Engineering, Edmonton, AB T6N 1E4, Canada; Tegbir.singh@innotechalberta.ca (T.S.); tonya.wolfe@innotechalberta.ca (T.W.)

**Keywords:** laser powder bed fusion (LPBF), benchmark test artifact, numerical simulations, dimensional metrology, geometric dimensioning and tolerancing (GD&T)

## Abstract

The precision of LPBF manufactured parts is quantified by characterizing the geometric tolerances based on the ISO 1101 standard. However, there are research gaps in the characterization of geometric tolerance of LPBF parts. A literature survey reveals three significant research gaps: (1) systematic design of benchmarks for geometric tolerance characterization with minimum experimentation; (2) holistic geometric tolerance characterization in different orientations and with varying feature sizes; and (3) a comparison of results, with and without the base plate. This research article focuses on addressing these issues by systematically designing a benchmark that can characterize geometric tolerances in three principal planar directions. The designed benchmark was simulated using the finite element method, manufactured using a commercial LPBF process using stainless steel (SS 316L) powder, and the geometric tolerances were characterized. The effect of base plate removal on the geometric tolerances was quantified. Simulation and experimental results were compared to understand tolerance variations using process variations such as base plate removal, orientation, and size. The tolerance zone variations not only validate the need for systematically designed benchmarks, but also for tri-planar characterization. Simulation and experimental result comparisons provide quantitative information about the applicability of numerical simulation for geometric tolerance prediction for the LPBF process.

## 1. Introduction and Background

According to ISO/ASTM 52900:2015 [1], additive manufacturing (AM) is defined as the “process of joining materials to make parts from 3D model data, usually layer upon layer, as opposed to subtractive manufacturing and formative manufacturing methodologies.” AM is also commonly known as “3D printing”. AM was developed in the early 1980s and has shown great promise and the ability to produce complex and custom geometrical shapes from a wide range of materials [2]. Common AM processes are material extrusion, vat photopolymerization, directed energy deposition, and laser powder bed fusion (LPBF) [3]. The LPBF process for metals uses a laser as a source of energy to fuse layers of metal powder. The laser melts a defined path on each layer of the deposited metal powder. The bed then moves down, and another layer of metal powder with a thickness of 20–100 µm is deposited. This process is repeated until the part is completely manufactured. No binders are used in this process. The process can produce fully dense metal parts ready for industrial use and has limited post-processing needs. It is a widely used metal AM process with various applications in the automotive, aerospace, and biomedical industries [4]. Although AM has made the transition from research labs to industry in the last decade, various challenges, such as geometric tolerance control of manufactured parts, low repeatability, and controlling deviations due to residual stresses still need to be addressed [2]. To define the challenges, the output characteristics of AM parts must be understood and can be categorized as follows:

- Mechanical characteristics, such as tensile, compressive, flexural strength;
- Material characteristics, such as microstructure and porosity;
- Surface characteristics, such as surface roughness and wear properties;
- Geometric characteristics, such as dimensional accuracy and geometric tolerances.

These output characteristics are dependent on various process parameters, such as layer thickness, scan strategy, and part orientation. Standardized procedures have already been established for mechanical, material, and surface characteristic testing and quantification [5,6,7]; however, standardized methods for quantification and assessment of geometric characteristics are still in their infancy for metal AM [8,9]. Like any other manufacturing process, additively manufactured parts also have deviations and errors compared to the input CAD data. Understanding these deviations or variations is critical in engineering design and manufacturing. For example, a higher frequency of part rejections is typical in precision applications, as a high degree of geometric control is challenging. Variations in a final part’s geometry occur due to process physics, various process parameters, and manufacturing conditions. Resulting “geometric deviations” or “geometric errors” need to be quantified and assessed in advance to ensure the required dimensions and geometric tolerances of the AM part are met.

To date, methods for geometric tolerance assessment of AM processes are not yet completely defined or standardized. Experimental methods based on geometric benchmark test artifacts, or GBTAs [10,11], aim at providing quantitative information about various geometric tolerance characteristics. A brief comparison of common GBTAs available in the literature is presented from a geometric tolerance perspective in Table 1. The features of the manufactured GBTAs are used to characterize different dimensional and geometric tolerances [12,13,14,15]. The GBTA experiments are followed by ranking and optimizing manufacturing parameters that influence the geometric tolerances, and this information is used to optimize these parameters for the actual manufacturing. For example, Shahrain et al. [16] varied 13 process parameters of a fused deposition modeling printer and, based on the experimental results for flatness and cylindricity, the parameters were ranked and optimized to meet the required geometric tolerance of subsequent prints. References [12,13,17,18,19,20,21,22] provide similar studies, where the geometric tolerances of manufactured GBTAs are characterized.

Even though these studies provided a great starting point for further study, their application in predicting geometric tolerances for other parts manufactured on the same machine is questionable. This is due to the lack of features in the GBTA to quantify certain geometric dimensioning and tolerancing (GD&T) characteristics as per ISO 1101 [23]. Such as step cylinders for concentricity, parallel flat features for parallelism, and so on. The inability of available GBTAs to provide accurate and reliable information about the geometric tolerance of actual parts makes them unsatisfactory for geometric quality characterization. A comparative study of various GBTAs tested on LPBF and the corresponding geometric tolerances is shown in Table 1. The comparison shows that each GBTA has characterized only some of the geometric tolerances, and those characteristics are not quantified in all three principal planar directions. The removal of support structures and the base plate is also not considered during GBTA design or measurements. Moreover, numerical simulation tools were not used to conduct geometric tolerance predictions for the GBTAs to be manufactured.

The comparative study highlights state-of-the-art GBTA design limitations to characterize critical GD&T, as well as other crucial geometric quality parameters, such as the minimum feature size and fit for assembly. Most of these experimental methods are not conducted on systematically designed benchmarks, lack proper design for the experiments, are based directly on application geometry, and are trial-and-error based. As such, multiple repetitions are required with various parametric changes, some of which do not actually add to any geometric information and eventually add up to the increasing costs. Thus, for very costly metals and large build volumes, repeated benchmark experimentation is not feasible. Significant limitations of the various benchmark artifacts present in the literature are:

- Inability to provide the geometric evaluation of major GD&T characteristics;
- Lack of features aligned with x, y, z directions for a robust single setup, tri-planar characterization;
- Lack of variation in feature size, specifically for medium to larger mechanical parts;
- No consideration of the effect of removal of the base plate towards the design and measurement phase or consideration of a very thick base feature not capable of capturing base surface warping effects;
- Inclusion of repetitive and/or redundant features that result in material and measurement time wastage;
- Artifact feature size incompatible with available measurement equipment;
- Lack of numerical simulation tools for predicting the geometric tolerances before actual manufacturing.

The solution is to systematically design GBTAs and conduct only minimal required experimentation for geometric tolerance characterization purposes to obtain useful data for quality characterization of the metal AM process and for prediction purposes. A “feature-based methodology” [29], which links primitive geometric features to GD&T characteristics, and that enables straightforward and systematic design should be used. The AM standard ISO 17296-3 [30] outlines the geometric characteristics that need to be measured, quantified, and controlled to define the geometric properties of an AM part:

- Size, length, angle dimensions, and dimensional tolerances;
- Geometrical tolerancing (deviations in form, orientation, and position).

The above-mentioned geometric characteristics are further standardized by geometric dimensioning and tolerancing (GD&T). The ISO 1101: Geometrical Product Specifications [23] standard defines GD&T in detail. GD&T characteristics enable the systematic quantification of form, location, and orientation tolerances of the features of a part. The 14 GD&T characteristics in ISO 1101 are explained briefly along with their drawing symbols in Table A1 of Appendix A. The generic guidelines outlined in ISO/ASTM 52902:2019 [31] for test artifact design should be followed carefully. Any new GBTA should quantify all the major GD&T characteristics in the tri-planar directions. Further, to minimize material and manufacturing time, GD&T estimation should be conducted using numerical simulations instead of experimental GBTA fabrication and characterization. Numerical simulation tools are rarely used to estimate GD&T and, as a result, the reliability of these tools to predict GD&T has not yet been assessed. Most research work is focused on deviation simulations, residual stress simulations, microstructure simulations, and topology optimization [32,33,34]. There is little to no research on the use of macro-scale finite element simulations to predict the geometric tolerances of LPBF parts according to the ISO 1101 standards. Thus, along with experimental datasets, a framework for simulation-based geometric tolerance prediction and analysis is also required.

This article aims to present a new GBTA design with a “feature-based methodology” [29], linking primitive geometric features to the GD&T characteristics and quantifying all the major GD&T characteristics in the tri-planar directions. GBTA will be used, not only to quantify GD&T, but also to assess the reliability of the proposed methodology to quantify GD&T based on numerical simulation tools. The aim is that, in the future, GD&T can be assessed using numerical simulations with the proposed GBTA being used for model calibration and verification. The detailed design process is explained in Section 2. It enables a generic quality characterization of the LPBF process under consideration from a GD&T perspective. The corresponding experimental plan and the measurement procedures are explained in Section 3. Section 4 outlines the numerical simulation process, which is used to extract deviated geometries, with and without a base plate, for a virtual GD&T characterization for comparison with the experimental results. Finally, the experimental and simulation results are presented in Section 5, followed by conclusions in Section 6.

## 2. Geometric Benchmark Test Artifact Design

The geometric benchmark test artifact (GBTA) was designed to fulfill two major objectives:

- To perform the tri-planar GD&T quantification of the metal AM process;
- To study the effect of base plate removal on GD&T characteristics.

To achieve the first objective, all major GD&T characteristics were quantified on a range of features and sizes on the GBTA. Also, the feature orientation was selected to enable the three principal planar directions for the tri-planar normative quantification of the AM process in consideration. Given the orthotropic, layer-by-layer fabrication of the AM process, and the coupled process physics, it is essential to consider tri-planar repeatability as the mere assumptions of tolerance band applicability across different axes is not valid. The features on GBTA are selected according to the feature-based GBTA design methodology [29], which gives systematic guidelines for selecting features according to the geometric tolerance characterization requirement. The ability to manufacture minimum feature sizes and overhangs was also considered. To fulfill the second objective, i.e., to characterize the effect of removing the base plate, GD&T quantifiers were measured before and after removing the base plate.

Features of the GBTA were selected based on the GD&T requirements for the normative quantification of the geometric quality characteristics of the metal AM process. The bounding box size of the GBTA was decided based on the build volume of the LPBF system on which it had to be manufactured. Since the build volume was 250 × 250 × 300 mm, the size of the GBTA base was kept at 200 × 200 mm and then features were spread on that surface. In the case of LPBF, the raster direction changes with a change in orientation of the part in the AM machine. Therefore, the orientation of the part has a significant effect on the geometric properties of the features. There are two different ways that this variation can be studied and characterized:

- Printing the features in different orientations on the same GBTA;
- Printing the complete GBTA in different orientations.

**Figure 1:**
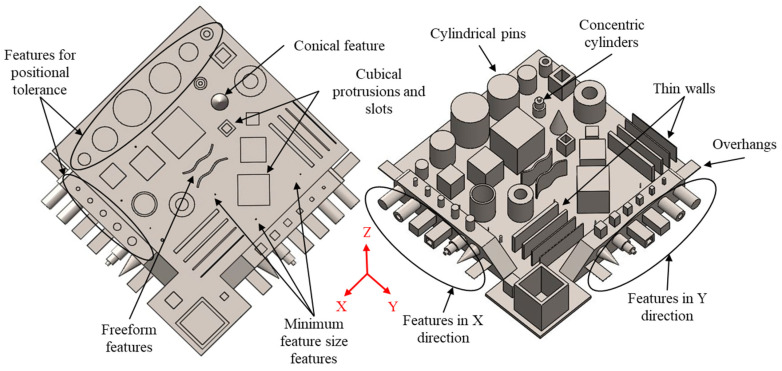
Nominal GBTA design and corresponding features. (**Left**) Top view and (**Right**) isometric view.

The former method is used in this study because, for the latter method, the support structure design for the features in different orientations can vary significantly and, as a result, it would not allow for a robust measurement and comparison of the benchmark artifacts samples. Therefore, the GBTA used in this study was built by printing features in different orientations on a common base. The features to be present on the GBTA base are shown in Figure 1 and are explained below:

- Cuboids: Hollow and solid cuboids selected to quantify straightness, flatness, perpendicularity, and parallelism. Since these are large volume features, the effect of the process on the deviations will be different compared to small volume features, such as thin walls.
- Thin walls: Thin walls of varying dimensions to quantify straightness, flatness, and the effect of residual stresses on the GD&T quantifiers for thin features.
- Cylindrical features: Solid and hollow cylindrical features placed with axes in all three principal directions (X, Y, and Z) to quantify circularity and cylindricity.
- Axially stacked cylinders: Stacked cylinders for measuring concentricity and runout in the X, Y, and Z directions.
- Conical features: For measuring the circularity of features other than cylindrical forms.
- Positional features: Features positioned at specified distances from each other to find positional tolerance zones of features.
- Profile: Profile features with curves to quantify the profiles of a surface and of a line tolerance.
- Minimum size features with diameters and external sizes ranging from 0.5 to 2 mm. This will help to check the ability of the metal AM process in consideration of printing minimum-sized features as well as the effect of the size on the tolerance.
- Thin overhangs: Thin overhang features to understand the effect of support and base removal on thin and fragile features.

**Figure A1:**
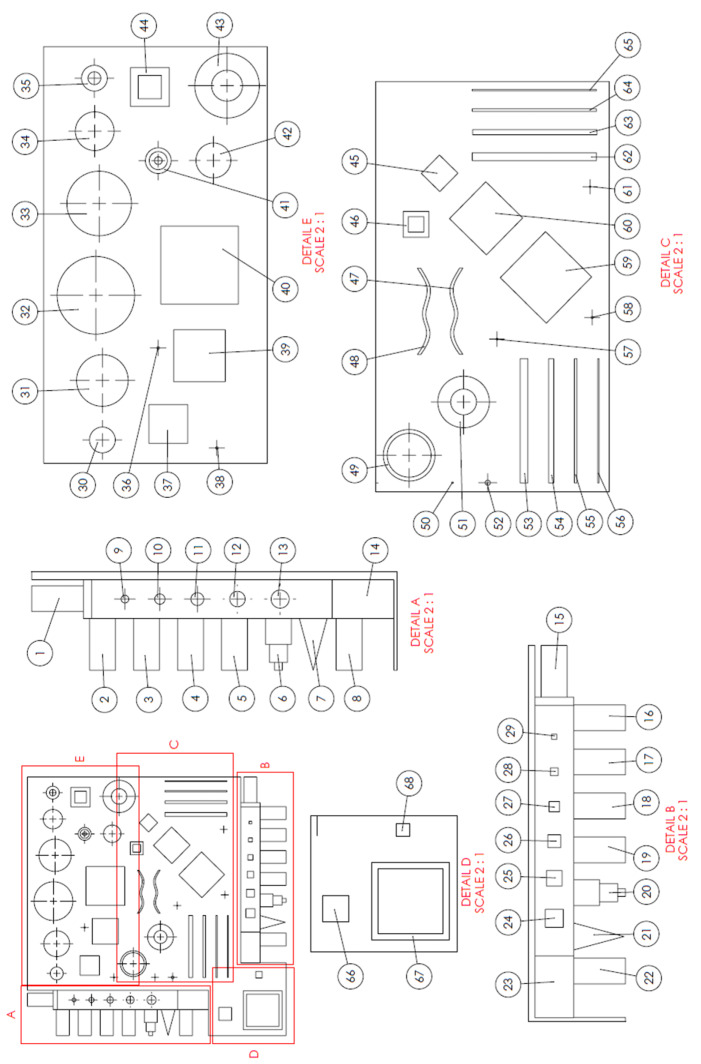
GBTA drawing depicting all the features along with their numbering.

In addition to the GD&T specifications and the other geometric quantifiers, the design needed to take into account the metal AM process constraints. The GBTA features were placed such that the maximum area on the base plate was covered to understand the spatial variations. The effect of variations in terms of feature size on the geometric tolerances is also an important aspect to consider, as the size tolerances significantly influence the design of metal AM in precision applications. The features are shown and explained in greater detail in Figure A1 and Table A2 of Appendix B, showing the numbering of the features on the GBTA, and the relevant geometric tolerances of the features. Complete drawings with size dimensions, location dimensions, and geometric tolerances are also provided in Supplementary Data.

## 3. Experimental Procedure

### 3.1. GBTA Manufacturing

Three newly designed GBTAs were manufactured using an AM250 LPBF machine (Renishaw PLC, Wotton-under-Edge, Gloucestershire, UK) [35]. The STL file of the GBTA was converted to a sliced file by specifying the process parameters, such as layer height, hatch style, and support structure, using the manufacturer-provided software (Renishaw QuantAM 2018). The process parameters were decided as per previous experience and manufactured using best practices. The process parameters for the manufactured parts were kept constant to investigate the variations in the process and are listed in Table 2.

**Figure 2:**
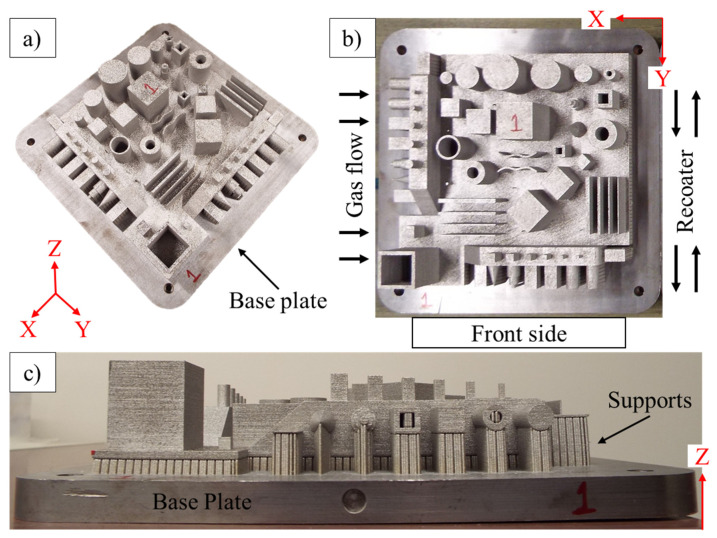
Example of manufactured sample: (**a**) Isometric view of the GBTA, (**b**) top view of the manufactured GBTA showing the “front side” of the LPBF system, gas flow direction, and recoater direction, and (**c**) side view of the manufactured GBTA showing the depicting and the base plate.

The build chamber was sealed, and argon gas was introduced to maintain an inert environment for laser-based manufacturing. Throughout the build process, the oxygen level was kept at less than 0.1%. The constant argon gas flow acted to keep the laser lens clean, reduce any oxygen or nitrogen entrapment, and moderate the vapor flow of the processed metal powder. Stainless steel (SS 316L) grade powder from Renishaw^TM^ (Wotton-under-Edge, Gloucestershire, UK) was used as a raw material for manufacturing. Upon completion of the build, the part was left in the chamber to cool down to room temperature for approximately 2–3 h and then removed. One of the manufactured samples, as manufactured and attached to the build plate, is shown in Figure 2. To understand the as-built geometric properties of the GBTA, the manufactured samples were assessed without any kind of post-processing.

### 3.2. Measurement Procedure

**Figure 3:**
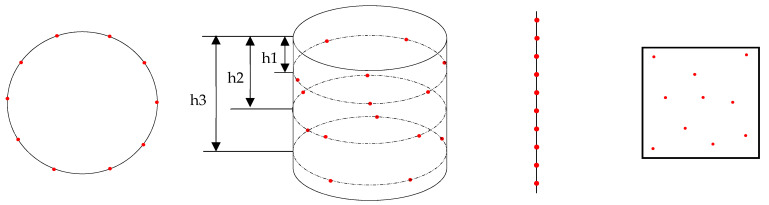
Measurement strategies for different features. The red dots represent the locations where the CMM probe recorded the measurement data.

The features on the manufactured samples were measured for various GD&T characteristics using a Mitutoyo Crysta-Plus M443 (Mitutoyo, Kawasaki, Japan) coordinate measuring machine (CMM). The CMM has a resolution of 0.5 µm. For measurements with the base plate, the GBTA was stationed on the CMM table with fixtures to restrict its movement. The measurements were conducted keeping in mind the tight tolerance zones of the LPBF process under consideration, so reducing measurement variability was a priority. For each characteristic measurement, at least ten data points were taken using the CMM and each measurement was repeated thrice to achieve repeatable readings. For one flatness tolerance zone of a feature, ten data points were collected from the surface to calculate it. The measurement process for this study was divided into four major geometric categories, i.e., circle, cylinder, line, and surface. The procedure adopted for the measurement for each geometry is listed below [36]:

- Circle: Measured using 10 equally spaced points around the circular cross-section.
- Cylinder: Measured using a total of 15 points with 5 points, equally spaced, distributed at three cross-sections of the cylinder at heights h1, h2 and h3 (see Figure 3).
- Line: Measured using 10 points, equally spaced, along the length of the line to cover at least 90% of the length of the line.
- Surface: Measured using 10 points with their random distribution over the surface to cover the maximum area of the surface.

The schematics of the measurement strategies are shown in Figure 3. The red dots represent the locations where the CMM probe recorded the measurement data.

The method of least squares was used to define planes and cylinders in the CMM for computing the GD&T tolerance zones. Two coordinate systems were defined to perform the measurements of the GBTA in the CMM. One is the coordinate system of the CMM itself, which defines the motion of the CMM probe in three directions, referred to as the X, Y, and Z directions. The second is the part of the coordinate system that is defined using the GBTA. It is the orthogonal frame of reference that defines the orientation of the features with respect to each other and is used for computing the orientation- and location-based geometric tolerances. The part coordinate system is shown in Figure 2. To eliminate ambiguity, both frames were aligned with each other on the same axes (X–X, Y–Y, and Z–Z) with each other. In the CMM, the surface–line–line method was used to set the part coordinate system [37]. The part coordinate system is depicted in Figure 2. The top surface of the base of the GBTA, on which most of the features are built, is defined as the XY plane. The features categorization for measurements, as per the GD&T, is shown in Table 3. The results are discussed in Section 5 according to the GD&T characteristic.

**Figure 4:**
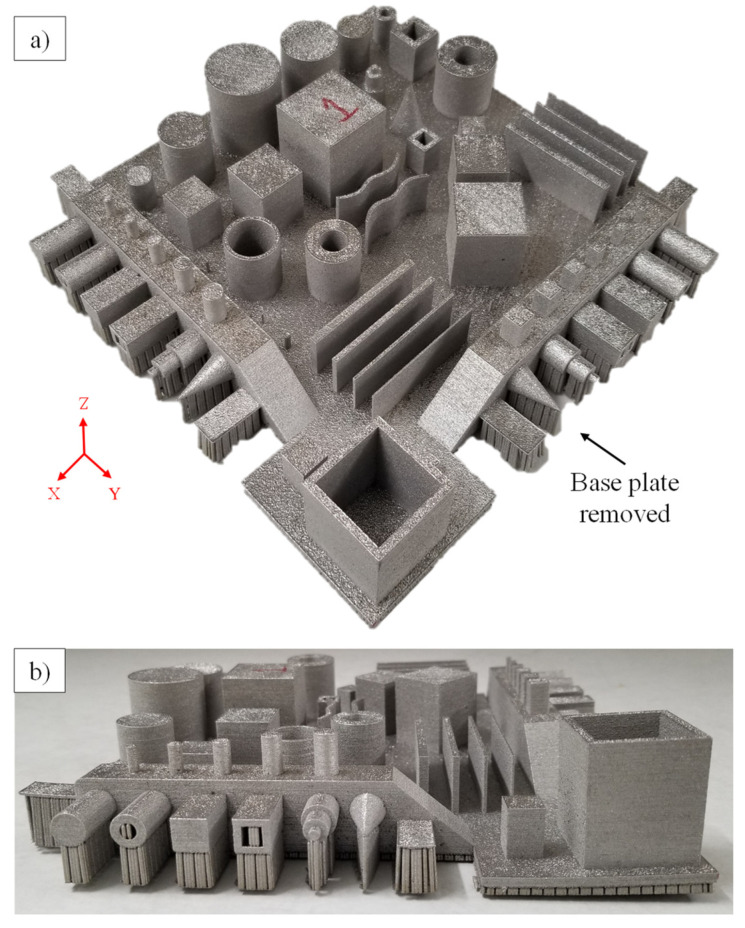
GBTA after the removal of the base plate: (**a**) Isometric view and (**b**) side view showing warped features.

For the features with support structures, such as features 1–8, the area covered by the support structures was excluded from the measurements. The supports were kept intact to make sure that the features were hinged on the base plate when the measurements were performed. For the second round of measurements without the base plate, first, the base plate was removed from the GBTA using a bandsaw. The support structures were also removed manually using hand tools. The GBTA, after the removal of the base plate and support structures, is shown in Figure 4. In addition, for fixing the GBTA after the removal of the base plate on the CMM table, special fixtures were used. These fixtures were designed according to the GBTA dimensions and manufactured using polymer 3D printing.

## 4. Numerical Simulation

A numerical simulation of the GBTA was conducted to understand the effects of temperature and stress fields on the specific planar GD&T characteristics, as well as to assess the reliability of the thermo-elastic finite element model implemented in Netfabb Local Simulation (Autodesk Inc., San Rafael, CA, USA) for GD&T quantification [38]. First, the part and the support structures were imported into Netfabb Local Simulation. Then, a process parameter file (PRM) was generated by conducting a micro-scale simulation using the experimental parameters for the SS 316L material. The process parameters used were the same as those used in the experimental study and are described in Table 2. The simulation parameters are shown in Table 4. The temperature-dependent material properties of SS 316L were based on data from References [39,40]. The PRM file was used to conduct a macroscale simulation of the GBTA part [41,42]. The boundary conditions for the macroscale model were as follows:

- Mechanical boundary conditions: The bottom nodes of the base plate were fixed, i.e., zero displacements was imposed in all directions. At the other boundaries, a zero normal stress boundary condition was used.
- Thermal boundary conditions: A convective heat transfer boundary condition was considered between the powder and the environment. A heat loss coefficient value of 20 W/m^2^/K was used [43].

**Figure 5:**
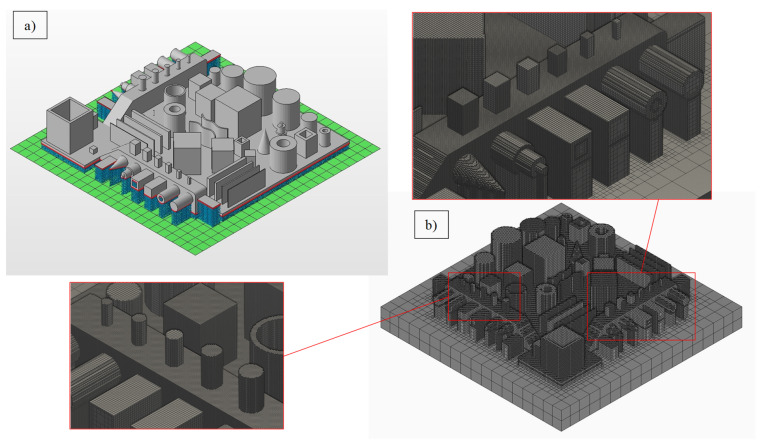
(**a**) GBTA with supports (in blue) and (**b**) layer-based adaptive coarsening mesh.

**Figure 6:**
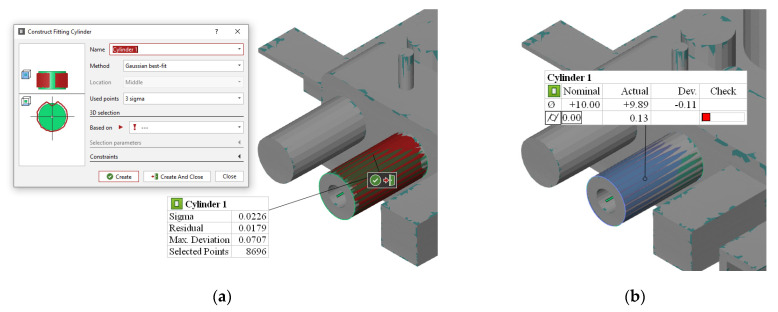
Extraction of GD&T: (**a**) construction of fitting feature on deviated STL and (**b**) extraction of GD&T characteristics based on the constructed feature.

The input STL, along with supports, is shown in Figure 5a and the meshing strategy is shown in Figure 5b. After a solution was obtained, the predicted displacement field was used to construct a deviated GBTA part. The deviated STL files were imported to GOM Inspect (GOM GmBH, Braunschweig, Germany) [44] for extracting GD&T quantifiers for a one-to-one comparison with the experimental results. Figure 6 shows the process of constructing fitted features on the deviated STLs from simulations and extracting the GD&T characteristics. The deviated STL file was first converted into a point cloud. Standard geometric shapes were then fitted into the individual features of the deviated point cloud, such as cylinder or plane. GD&T definitions were then used to find the tolerance zones on these fitted features. The GD&T results from the simulations (both with and without base plate) are discussed alongside experimental results in the next section for analytical comparison and physical interpretation.

## 5. Results and Discussion

The measurement data from the experiments and the predicted deformed geometry from simulations are used to extract the GD&T characteristics and are presented in the following sections. The data from the samples after removing the base plate are also presented to have a comparative analysis and discussion.

### 5.1. Form Tolerances

#### 5.1.1. Straightness

**Figure 7:**
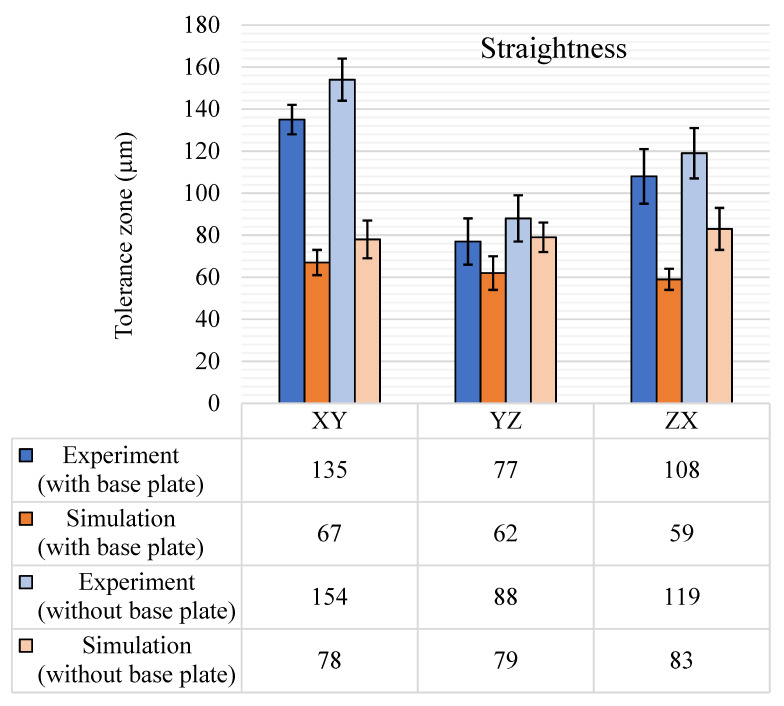
Straightness tolerance results (μm), with and without the base plate.

The straightness tolerance zones were computed for the features and categorized according to the feature’s orientation to the reference planes—XY, YZ, and ZX. Results for features parallel to reference plane XY are shown with bars labeled “XY” in Figure 7. A similar approach is used for YZ and ZX. For the experimental measurements, the sample space contained 13 features with a feature axis aligning with the XY plane and 8 for each of the YZ and ZX planes. For example, the first XY bar in Figure 7 shows the experimental results for the average tolerance zone for the 13 features aligned to the XY plane with the base plate intact on the GBTA.

Experimental results showed that the straightness tolerance was anisotropic with respect to the reference planes since the results were different in all three principal planar directions, along with variations in standard deviation. The straightness tolerance was higher in the XY planar direction because the measurements were performed on the as-built parts without any post-processing; therefore, the hatching effect cannot be ignored on this plane. The effect is increased by chessboard (or checkerboard) style hatching, in which the laser movement is multi-directional and creates a crisscross waviness on the surface. The hatching effect was maximum on surfaces parallel to the XY plane as it is the plane on which the laser moved while the part was built. In general, the straightness tolerance results increased by 15 to 20 µm in all three planar directions after the removal of the base plate, which was anticipated as the residual stresses were released after the base plate removal, which, in turn, increased the deviations. Even with a larger curvature on the base feature of the GBTA after the removal of the base plate, the form tolerances, such as straightness, were less affected, as they were independent of datums. The largest straightness tolerance zone was observed on a thin sheet (feature 9) on the XY plane. It was 158 µm with the base plate and 183 µm without the base plate. The large tolerance zone resulted due to the low thickness value, i.e., 500 µm, of the sheet, which led to larger deformations during the rapid cooling of the part throughout the process.

Figure 7 shows that the predicted straightness tolerances by numerical simulations. The simulation results closely followed the experimental results in the YZ plane; however, there is a dip in simulation results in the XY plane, which could be because the hatching phenomena was not properly captured in simulations. In all three directions, the overall tolerance band is 40 µm lower for both (with and without base plate) results and follows the same pattern. It is hypothesized that the reason for the major variation in the straightness tolerance of the XY plane was that the surface waviness due to hatching was not captured well by the simulations. The difference in results is in a similar range with the generic surface waviness values for LPBF-manufactured SS 316L parts [5]. The difference in the experimental results in the YZ and ZX planes was also not completely captured by the simulations. Since straightness is a form tolerance and the boundary conditions have a maximum effect on the form tolerances, the “uniform heat loss condition” could be one reason for this similarity in simulation results for the YZ and ZX planes. However, the standard deviations for the XY plane are almost the same for the experimental and numerical results, with and without base plate, i.e., only ±1 µm.

#### 5.1.2. Flatness

**Figure 8:**
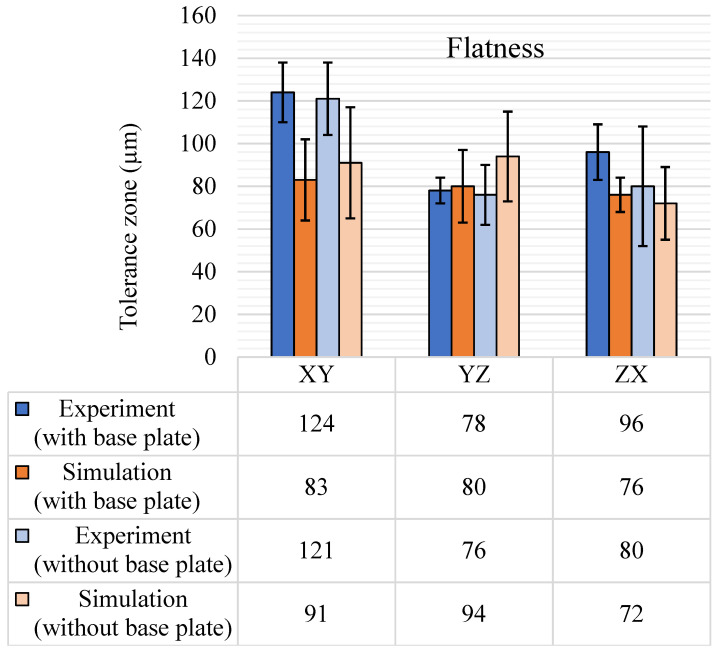
Flatness tolerance results (μm) with and without the base plate.

The sample space for flatness tolerance contained 20 features for the XY plane, 25 features for the YZ plane, and 24 features for the ZX plane. The flatness results for both experiments and simulations are shown in Figure 8, with a comparison of with and without base plate removal. Much like the straightness results, the anisotropy of the experimental results was observed for the flatness tolerance as well. The largest tolerance bands with and without the base plate were observed for the XY plane and the smallest for the YZ plane. Maximum tolerance bands are observed for the XY plane for both with and without base plate conditions. This is because the XY plane was the building plane, which also bore the effect of in-plane shrinkage during the building of the part. Further, the release of the residual stress with the removal of the base plate also affected the XY plane to the highest degree as the base feature of the GBTA was parallel to the XY plane, on which all the features were mounted. Since flatness tolerance was spread over the complete region of the feature, even minor curvatures and warpage due to the removal of the base plate affected the tolerance zone. A large standard deviation in the results for the ZX plane without the base plate was observed, i.e., a standard deviation of 56 µm. This could be due to the recoater direction, which was perpendicular to the ZX plane and affected the layer edges during the building, hence reducing the surface quality of the ZX walls.

Looking at the simulation results in Figure 8, an overall lower range of flatness tolerance is observed as compared to experimental results, except for the YZ plane. For both experiments and simulations, the standard deviation of the results increased with the removal of the base plate. This shows that with the removal of the base plate, some of the surfaces were warped to a greater extent, leading to a rise in standard deviation. For instance, features 4, 5, 18, and 19 show an approximate increase of 20 µm in flatness for the XY plane after the removal of the base plate as they were closer to the edge and were most affected by the warpage of the GBTA base feature. An interesting result to notice is the standard deviation of the ZX plane with the base plate, which reduces from 26 µm to 16 µm. The possible reasoning behind this could be that the features that had larger flatness tolerance bands experienced warpage in such a way after the base plate removal that the flatness values dropped.

#### 5.1.3. Circularity

**Figure 9:**
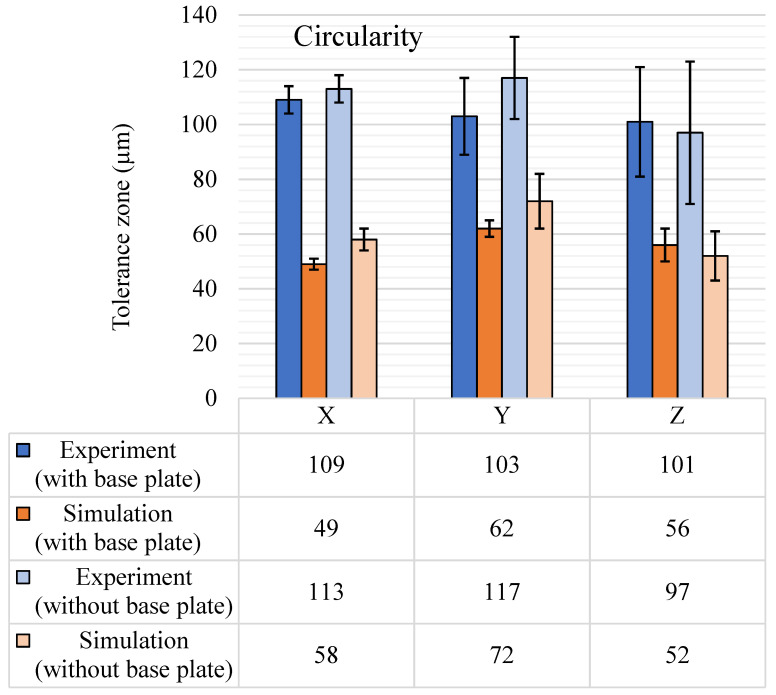
Circularity (μm) tolerance results, with and without the base plate.

Measurements were done for different cylindrical and conical features with their axes aligned to their respective reference axial directions—X, Y, and Z. The tolerance zones were computed for these categories of features, and the same are represented in the bar chart in Figure 9. The sample space contained six features each for the X and Y axes directions and 26 features for the Z-axis direction. The larger number of features in the Z direction varied in terms of size and volume to get a detailed account of the circularity variation in the principal build axis, i.e., the Z-axis.

**Figure 10:**
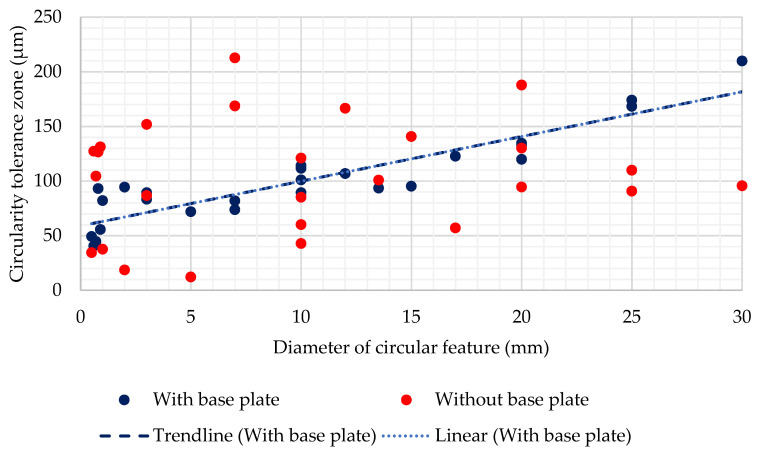
Diameter vs. circularity (experimental).

For the experimental results, the circularity tolerance zone with the base plate intact was around 104 µm for all three axial directions. However, the standard deviation was highest for the Z-axis, i.e., 40 µm, and without the base plate, the standard deviation increased to 52 µm. The large standard deviation observed in Z-axis was due to the removal of the base plate and because a large number of features were characterized in the Z-axis direction. However, since circularity is measured on features of revolutions, the change in the diametric value of the feature was also correlated to circularity tolerance. Trends for circularity vs. diameter in the Z-axis direction are shown in Figure 10. Similar results were observed for experimental circularity tolerance after the removal of the base plate.

For circularity with the base plate, a clear relationship was observed, i.e., the circularity tolerance zone increased with the increase in diameter. It was also depicted by a linear trend line with an R-squared coefficient value of 0.8272. However, for circularity results after removing the base plate, no particular trend was observed. The most reasonable explanation is the uneven distribution of the residual stress led to variations in the deviation. Since with the change in the diameter, the surface area of the feature changed, which led to a change in residual stress and the resulting deviations or the tolerance zone.

In the simulation results, an overall mean shift of around 40 µm was observed in all directions for results with and without the base plate. The major reason for this mean shift could be the generic under-prediction observed in the deviation simulation and the inability of the simulation to capture meso-level surface variations. Apart from this mean shift and low standard deviation, the simulation results followed a very similar trend to the experimental results, especially for results after removal of the base plate.

#### 5.1.4. Cylindricity

**Figure 11:**
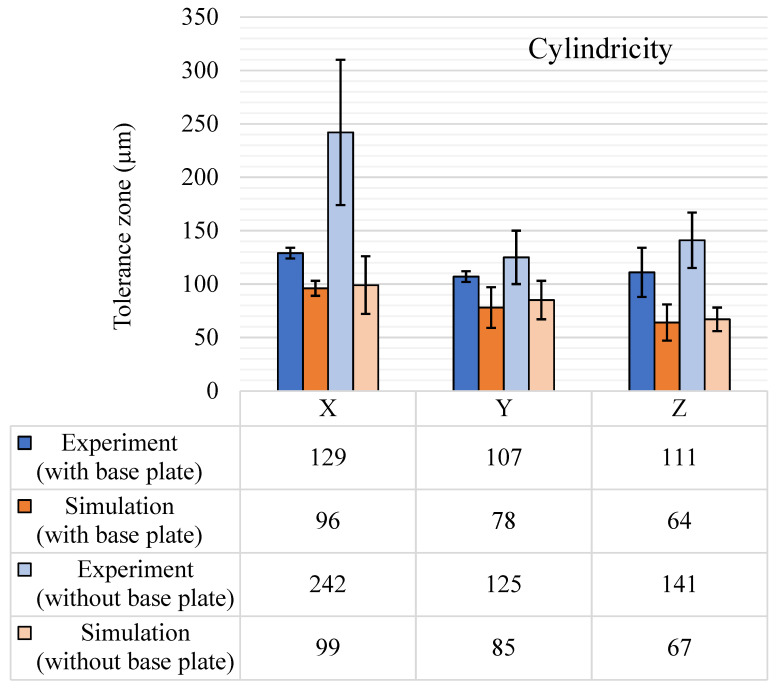
Cylindricity (μm) tolerance results, with and without the base plate.

**Figure 12:**
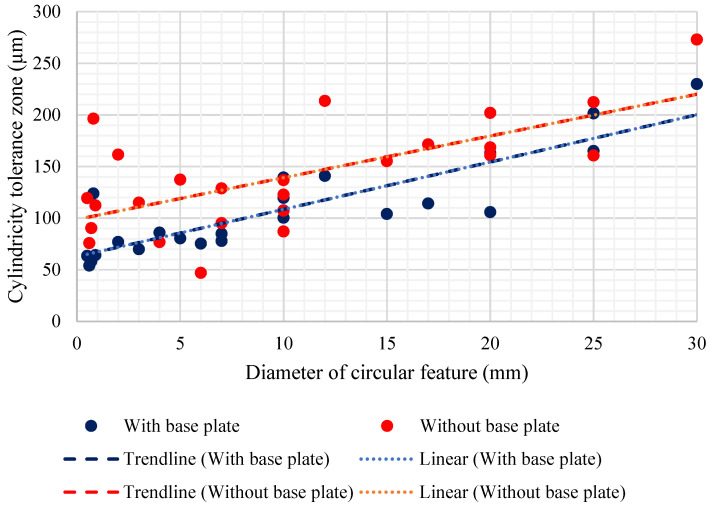
Diameter vs. cylindricity (experimental).

Cylindricity tolerance results are shown in Figure 11. The sample space contained six features each for the X and Y direction and 25 features for the Z direction. The diameter of the cylindrical features varied from 0.5 mm to 30 mm. Unlike circularity, the directional results after the removal of the base plate were not in a similar range for the three principal axial directions. The results in axial direction X showed the highest peak in the experimental cylindricity tolerance zone, as well as a standard deviation hike. One reason for this huge variation is the large support structures, which decreased the dimensional fidelity of the features, and along with the residual stress release, another reason could be the cantilever effect due to the nature of the geometric forms of the feature [45]. The effect of the diameter was also studied for the cylindricity tolerance zones, and the results are shown in Figure 12. A linear trend is observed with the cylindricity tolerance zone, which gets wider with an increase in the diameter of the cylindrical feature. Even with a few outliers with small diameter features, both sets of results (with and without the base plate) showed a similar trend. The trendline (with base plate) has an R-squared coefficient value of 0.7734, and for the trendline (without the base plate) the R-squared coefficient value is 0.5607. The results without the base plate were offset with an additional 40–50 µm.

Simulation results for cylindricity with the base plate intact are close to the experimental results. Unlike the large rise of the cylindricity observed in the X-axis direction for the experimental results without the base plate, the simulation does not show any such anomaly. This could be because the surface defects induced by the removal of the support structures are not captured by simulations. Moreover, a rise in cylindricity for the features in the X direction (such as features 2, 3, and 6) is still observed in the simulation results, similar to experimental results, due to the removal of the base plate and the support structures. However, the rise in the cylindricity tolerance with the removal of the base plate is still on the lower side compared to the tolerance increase seen for other form tolerances. This is because cylindricity tolerance is based on the form of the cylindrical feature, but not on the orientation, and with the removal of the base plate, the maximum effect is seen on the orientation as compared to the form of the cylindrical features.

Overall, analysis of the results for the form tolerances shows a general increase in tolerance zones after the GBTA is removed from the base plate. It can directly be linked to the release of the residual stress within the GBTA. Additionally, the increase in standard deviation suggests a similar reason. Apart from this, the process parameters and laser-material interaction have a significant effect, which will become clearer when the results are analyzed for a set of process parametric variations. For now, it can be stated that for the selected process parameters and SS 316L material, the form tolerances are generally in the range of 50 to 130 µm with a few exceptions, such as the spike in cylindricity in the X-axis direction after removal of the base plate.

### 5.2. Orientation Tolerances

#### 5.2.1. Perpendicularity

**Figure 13:**
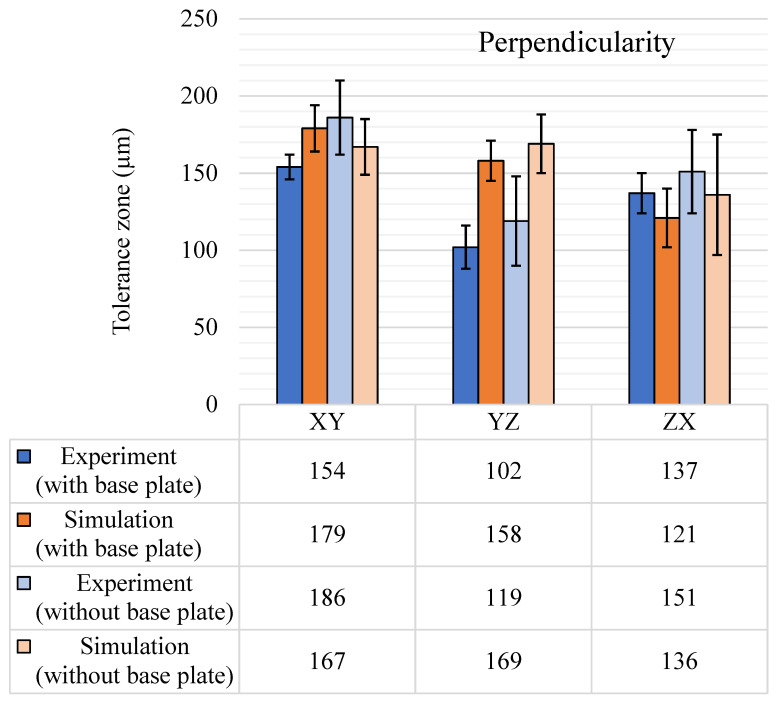
Perpendicularity (μm) tolerance results, with and without the base plate.

The GBTA contained 16 features for the XY plane and 18 features for both the YZ and ZX planes on which perpendicularity is measured. The perpendicularity tolerance zone results are shown in Figure 13. For experimental perpendicularity, the standard deviation increased by a factor of two when the base plate was removed. It increased to 54 µm from 24 µm. The maximum experimental tolerance bands were observed in the XY plane. The simulation results for perpendicularity were very close to those of the experimental results with few exceptions. The major variation consistently observed in the perpendicularity results was that the perpendicularity was minimum in the YZ plane for the experimental results, but for simulation results it was for the ZX plane. In addition, the simulation results in the YZ plane were higher than the experimental results, which was not the usual case in all other tolerance characterizations and simulation results. Since perpendicularity is an orientation tolerance, the flatness of the datum plane affected the tolerance zone, which was seen in both the experiments and the simulations. The variation in the flatness of the datum planes also propagated the tolerance zone to the perpendicularity results. This could be the major reason for the inconsistencies and variations in the results.

#### 5.2.2. Parallelism

**Figure 14:**
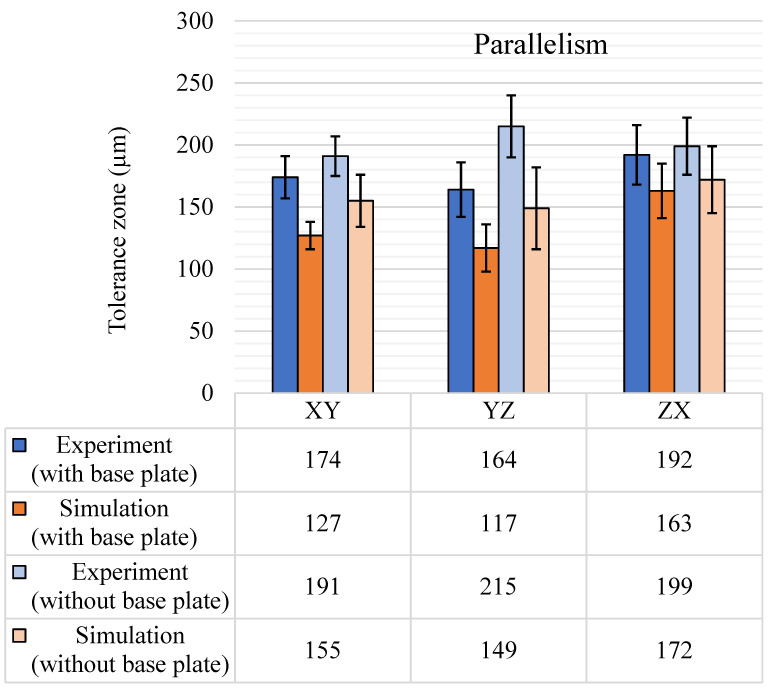
Parallelism (μm) tolerance results, with and without the base plate.

The sample space for parallelism tolerance contained 15 features for the XY plane and 13 features each for the YZ and ZX planes. Parallelism results from the experiments and simulations are shown in Figure 14. The experimental results show the anticipated increase of the tolerance zone after the removal of the base plate. The standard deviation values are different for each planar direction, but the average standard deviation for the three planes remained similar for both with and without base plate results. The results with the base plate for XY and YZ are comparable; however, there is a rise of around 20 µm in the ZX plane. The results without the base plate show the highest average tolerance zone of 215 µm for the YZ plane. In general, the small-sized features showed a larger parallelism tolerance zone with the base plate intact. However, after removing the base plate, the tolerance zone variations in the small features were less than the tolerance zone variations in the large features. The increase in the average tolerance zone after removing the base plate was due mainly to the larger size features as they are susceptible to more warpage and deformations with the release of stress. The simulation results showed a very similar trend for variations between the planes, especially for results with the base plate. The planar variation follows the same trend as the highest ZX plane tolerance zone with a similar standard deviation. However, the zone is 36 µm smaller than the experimental results. The same variations were not observed in the results without the base plate. In addition, the standard deviation for the results without the base plate increase by 14 µm from the experimental to simulation results.

#### 5.2.3. Angularity

**Figure 15:**
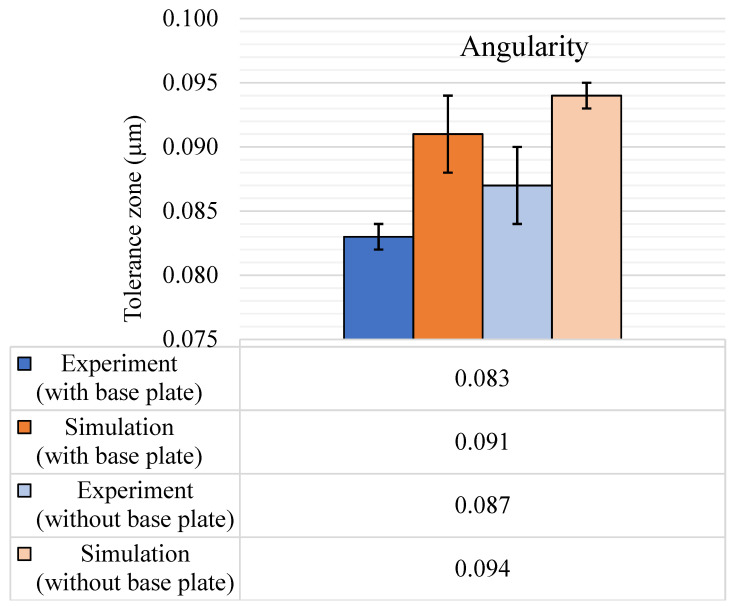
Angularity (μm) tolerance results, with and without the base plate.

Angularity measurements were done for two planar features at 40-degree angles to the XY plane and are shown in Figure 15. The experimental angularity increased after removing the base plate. For simulations, the rise in the tolerance zone after removal of the base plate was similar at 3 µm. However, the average tolerance zone was higher by 8 µm, i.e., at 91 µm with the base plate compared to 83 µm for the experimental value.

### 5.3. Location Tolerances

#### 5.3.1. Concentricity

**Figure 16:**
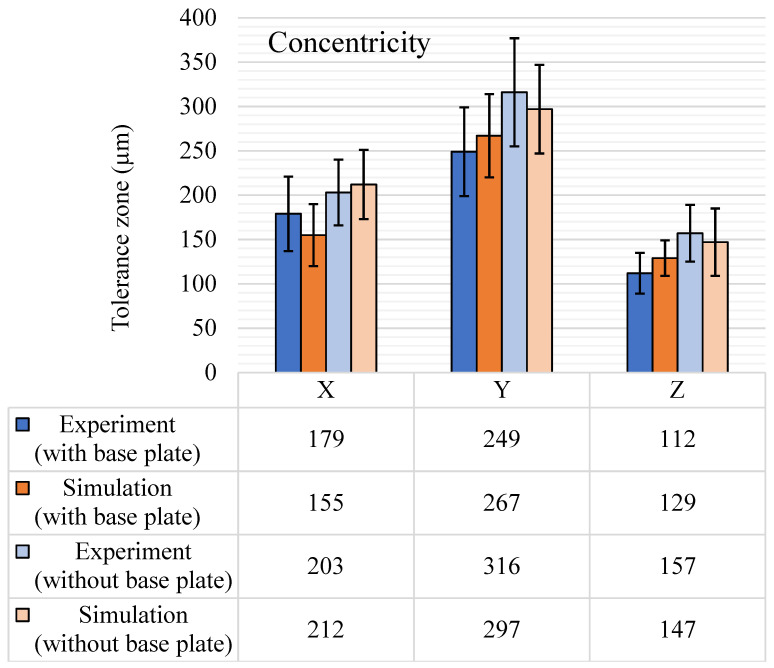
Concentricity (μm) tolerance results, with and without the base plate.

Concentricity tolerance is an important location tolerance for deciding the location of one cylindrical feature with respect to another and plays a major role during assembly operations. The concentricity results are shown in Figure 16. Experimental concentricity results for the base plate intact and without the base plate follow similar trends with a maximum tolerance zone in the Y-axis and a minimum in the Z-axis. It is worth noting that among all the GD&T characteristics discussed above, concentricity had the largest tolerance zones. This is due to the composite nature of concentricity tolerance, as it also depends on the form of the cylinders and the axial shift of the cylindrical features with respect to each other. Even with two features per axis and three measurements per feature, the standard deviation is large. The simulation results also follow suit with average concentricity tolerances being highest along the Y-axis and minimum along the Z-axis with an increase in the tolerance zone after the removal of the base plate. The standard deviations for the simulation results closely followed the experimental results.

#### 5.3.2. Position

**Figure 17:**
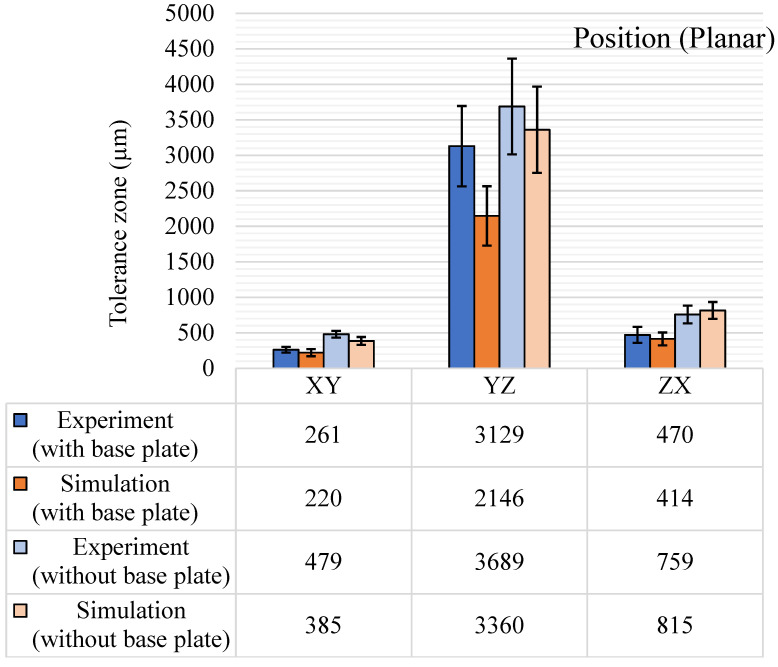
Position tolerance (planar) results (µm), with and without the base plate.

**Figure 18:**
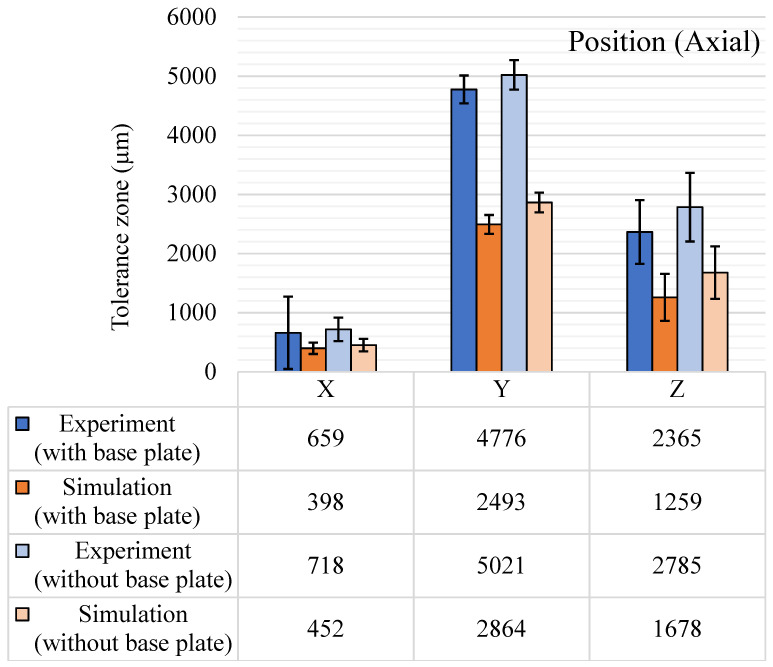
Position tolerance (axial) results (µm), with and without the base plate.

The GBTA features were positioned evenly on the base to study the position tolerance both for the planar and axial positions and the results are shown in Figure 17. The planar position variation was highest in the YZ plane for all results. The YZ plane is perpendicular to the recoater direction and the recoater movement and powder spread could be the most significant reason for these large tolerance zones. A similar trend was observed in the simulations as the simulation solver also considered the recoater direction and the recoater interference. The standard deviations also got wider for the YZ planar direction results along with a rise in the tolerance zone. For the axial position tolerance, cylindrical features were used across the entire GBTA and the results are shown in Figure 18. The experimental and simulation results are consistent in terms of variations across the axes. In the axial position, the Y-axis tolerance zone was highest for both experimental and simulation results. Simulation results have underpredicted the position tolerance for both planar and axial features. The standard deviations were much higher than any standard deviations observed for other GD&T characteristics, which is because the tolerance values are quite a bit higher than other tolerance characteristics for some of the positional features. The exact reasoning behind this significant shift is still unknown, but the most logical reason is the recoater direction effect and the micro-mirror orientations governing the laser beam.

### 5.4. Runout Tolerances

**Figure 19:**
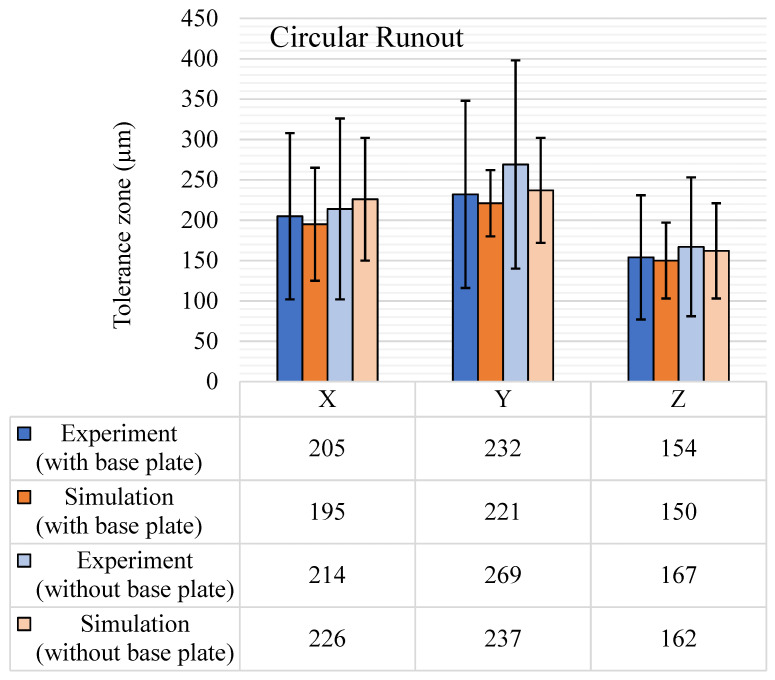
Circular runout (μm) tolerance results, with and without the base plate.

**Figure 20:**
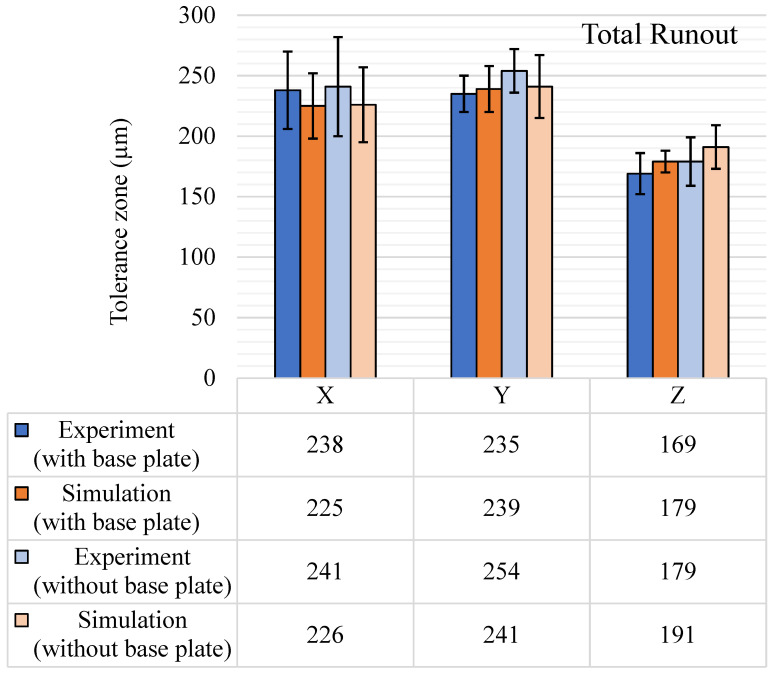
Total runout (μm) tolerance results, with and without the base plate.

Circular runout and total runout tolerance results from both experiments and simulations are shown in Figure 19 and Figure 20, respectively. Circular runout, in theory, is a more precise way of measuring circularity as it considers all the points on the periphery of the circular feature that is measured. This is why almost all the circular runout tolerance zones shown in Figure 19 are 50–100 µm more than the respective circularity tolerance results (Figure 9). The average tolerance band for experimental circular runout is 197 µm with the base plate intact. After the removal of the base plate, the average value reached 217 µm. The standard deviation also increased from 99 µm to 109 µm. The maximum runout was observed for the Y-axis features, and the same trend was observed in the simulation results. However, the simulation results for the circular runout showed a downward mean shift of around 10 µm in average tolerance values and the standard deviation was also reduced by at least 30 µm. The average change was mostly due to the drop in the values in the directions of the Y and X axes. For experimental results, due to the removal of the base plate, surface defects come into play, which is not the case in simulations. This effect is not significant in total runout results, as it considers the complete feature, and the effect diminishes compared to the tolerance zone of the complete feature. As shown in Figure 20, the results for total runout are quite a bit higher than the circular runout and even higher than concentricity results except for the Y-axis features.

For total runout, the experimental and simulation results were quite similar for the with and without base plate results, i.e., 214 µm and 224 µm, respectively. The standard deviation increased from 21 µm to 26 µm. Similarly, for the simulation results, the average tolerance values increased from 214 µm to 219 µm and standard deviation values rose from 18 µm to 25 µm. The trend was similar to the trend observed for circular runout and concentricity and the major reasons for this were the support removal and warpage due to residual stresses.

## 6. Conclusions and Future Scope

Geometric tolerance characterization is necessary for AM processes due to the inherent residual stresses during the build process that leads to variations in geometric tolerances. A new feature-based design of a geometric benchmark test artifact (GBTA) for LPBF is presented, which can characterize major geometric tolerance characteristics in three principal planar directions. The GBTA was manufactured using stainless steel (SS 316L) using a LPBF process and subjected to geometric tolerance measurements. The measurements were conducted on the GBTA in two stages: first with the base plate intact and then with the base plate removed. The geometric tolerance zones for features aligned with principal planar and axial directions were presented. The variations in the geometric tolerance zones with orientations and sizes were discussed.

Major conclusions from this research work are summarized below:

- The results not only justify the new GBTA design and its features, but also gives a quantitative outlook on the variation of the geometric tolerances according to orientation, sizes, and base plate condition;
- The results show that minimizing the residual stress and overall deviations do not lead to minimum tolerance zones for various geometric characteristics that dictate the functionality of the part;
- The circularity and cylindricity tolerance zones show a direct proportionality linkage to the diameter of the feature;
- The orientations of the features lead to a wide range of variation in geometric tolerance results, the average form tolerance increases after the removal of the base plate;
- The orientation and position tolerances also show an increase but, in some cases, the combined effect of the stress relief from removal of the base plate and the variation in the tolerance zone of the datum features minimize the overall tolerance variation;
- The results prove the need for directionality-based analysis of geometric tolerances and the need to consider the removal of the base plate;
- The simulation results are utilized to get an understanding of the tolerance variations and the effect of the residual stresses, with and without the base plate.

However, for specific applications, custom features and analysis are required to ascertain if given tolerance specifications are met. Moreover, since the simulation results are not in 100% agreement with the experimental results, this leads to the conclusion that more research is required to make sure that simulations can precisely predict the geometric tolerances for the LPBF process and can take into account the uncertainty of the process. It is hypothesized that the geometric tolerances will be affected by the selection of process parameters. Therefore, a process parameter optimization for specific geometric tolerances is should be performed after a normative benchmark analysis. The authors’ future work will be to create a training database for a machine learning algorithm that can predict variations in geometric tolerances with process parameter changes.

## Figures and Tables

**Table 1:** Comparison of selected GBTAs according to geometric tolerance characteristics.

Ref.	Geometric Tolerance Characteristics	Tolerance Directionality	Effect of Base Plate Removal Studied	Prior Numerical Simulation Conducted	Comments on GBTA Design
[12]	Straightness, flatness, roundness, cylindricity, perpendicularity, parallelism, profile, concentricity, position	Positive Z direction, XY plane, and few negative features in the Y direction	No	No	Widely accepted NIST standard test artifact (generic) Process-specific geometric evaluation requirement ignored Very thick base plate
[24]	Straightness, flatness, circularity	Positive Z direction and XY plane	No	No	Eight different samples regarded as one GBTA Manufactured in different batches (a potential source of deviation)
[25]	Flatness, squareness, parallelism, circularity	Positive Z direction and XY plane	No	No	The positional deviation is observed in all features Lack of process-specific pre-processing analysis
[26]	Straightness, flatness, roundness, concentricity, total runout	For flat features: XY plane For cylindrical features: positive Z-axis	No	No	Surface roughness and topographies measured Geometry linked to scan strategy Focussed on flat features only
[27]	Flatness, cylindricity, true position	Features in bi-planar directions	No	No	Parts stacked, not ideal for repeatable results Geometric tolerances not studied even when the features are present such as cylindricity
[28]	Straightness, flatness, roundness, cylindricity, perpendicularity, parallelism, angularity, profile, concentricity, position	Positive features in the bi-planar direction	No	No	Directional true position not studied Scalability issue Feature accessibility issue

**Table 2:** Machine parameters and manufacturing parameters for the experimentation.

Machine Settings	Value	Manufacturing Parameters	Value
Layer thickness	50 µm	Powder material	Stainless steel (SS 316L)
Laser power	200 W	Powder particle size	15–45 µm
Base plate size	250 × 250 × 15 mm	Hatching style	Chessboard style
Scan speed	600 mm/s	Hatch spacing	150 µm
Laser beam diameter	70 µm	Interlayer angle	67°

**Table 3:** Feature categorization for measurements based on GD&T characterization.

#	GD&T Characteristics	Features Categorization for Measurement
1	Straightness	Features with straight-line–flat surfaces of cuboids and thin sheets.
2	Flatness	Features with flat surfaces-cuboids, the top surface of cylinders.
3	Perpendicularity	Flat surfaces of cuboids and thin sheets with respect to a planar surface as a datum feature.
4	Parallelism	Features with flat surfaces parallel to a planar surface as a datum feature
5	Angularity	Angular features with respect to a planar surface as a datum feature.
6	True Position	Cylindrical and planar features with respect to a datum coordinate system.
7	Circularity	Cylindrical features–circular periphery on the outer surface of cylinders and hollow cylinders.
8	Cylindricity	Cylindrical features.
9	Concentricity	Stacked cylindrical features.
10	Circular run-out	Stacked cylindrical features.
11	Total run-out	Stacked cylindrical features.

**Table 4:** Simulation parameters.

Simulation Parameter	Value
Heat source absorption efficiency (%)	35
Analysis type	Thermal and mechanical
Structural plasticity calculation	After the part is cooled down and before removing the base plate
Mesh approach	Layer based
Maximum mesh adaptively levels	5
Coarsening generations	1
Layers per mesh element	20

## Data Availability

The raw/processed data required to reproduce these findings cannot be shared at this time as the data also form part of an ongoing study.

## Supplementary Materials

The following are available online at https://www.mdpi.com/article/10.3390/ma14133575/s1, A PDF file containing a set of drawings depicting the size dimensions and geometric tolerances of the geometric benchmark test artifact (GBTA).

## Appendix A. GD&T Characteristics

**Table A1:** GD&T characteristics, symbols and corresponding descriptions.

GD&T Characteristic Symbol	Control Type	Name	Summary Description
	Form	Straightness	Controls the straightness of a feature in a relation to its perfect form
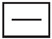
	Form	Flatness	Controls the flatness of a surface in relation to its own perfect form
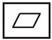
	Form	Circularity (Roundness)	Controls the form of a revolved surface in relation to its perfect form by independent cross-sections
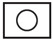
	Form	Cylindricity	Circularity’ applied to the entire revolved surface
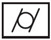
	Orientation	Perpendicularity	Controls the orientation of a feature that is nominally perpendicular to the primary datum of its datum reference frame
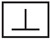
	Orientation	Parallelism	Controls orientation of a feature that is nominally parallel to the primary datum of its datum reference frame
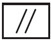
	Orientation	Angularity	Controls orientation of a feature at a specific angle in relation to the primary datum of its datum reference frame
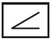
	Location	Concentricity	Controls concentricity of a surface of revolution to a central datum
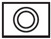
	Location	Position	Controls the location and orientation of a feature in relation to its datum reference frame
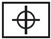
	Location	Symmetry	Controls the symmetry of two surfaces about a central datum
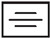
	Profile	Profile of a line	Controls the size and form of a freeform feature. Additionally controls the location and orientation when a datum reference frame is used
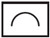
	Profile	Profile of a surface	Profile of a line’ applied to the complete feature surface
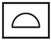
	Runout	Circular Runout	Controls circularity and coaxiality of each circular segment of a surface independently about a coaxial datum
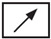
	Runout	Total Runout	Controls circularity, straightness, coaxiality, and taper of a cylindrical surface about a coaxial datum
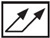

## Appendix B. GBTA Features

**Table A2:** GBTA features description as per the numbering.

GD&T Characteristics	Plane/Axis	Features
Flatness	XY	1, 4, 5, 8, 13, 15, 18, 19, 22, 24, 30, 31, 32, 33, 37, 39, 40, 45, 60, 66, 68
	YZ	2, 4, 18, 19, 24, 37, 39, 40, 44, 62, 63, 64, 65, 66
	ZX	4, 5, 16, 18, 24, 37, 39, 40, 44, 53, 54, 55, 56, 66
Straightness	XY	1, 4, 5, 8, 15, 18, 19, 22, 37, 39, 40, 53, 62
	YZ	24, 37, 39, 40, 62, 65, 66, 67
	ZX	24, 37, 39, 40, 53, 56, 66, 67
Parallelism	XY	4, 5, 8, 13, 18, 22, 24, 30, 31, 32, 37, 39, 40, 60, 66
	YZ	2, 4, 18, 19, 24, 37, 39, 40, 62, 63, 64, 65, 66
	ZX	4, 5, 16, 18, 24, 37, 39, 40, 53, 54, 55, 56, 66
Perpendicularity	XY	4, 5, 8, 18, 19, 22, 24, 37, 39, 40, 66
	YZ	24, 37, 39, 40, 62, 63, 64, 65, 66
	ZX	24, 37, 39, 40, 53, 54, 55, 56, 66
Angularity		14, 23
Circularity	X	2, 3, 6B, 6M, 6T, 7
	Y	16, 17, 20B, 20M, 20T, 21
	Z	9, 11, 13, 30, 31, 32, 33, 34, 35, 36, 38, 41B, 41M, 41T, 42, 43O, 43I, 49O, 49I, 50, 51O, 51I, 52, 57, 58, 61
Cylindricity	X	2, 3, 6B, 6M
	Y	16, 17, 20B, 20M
	Z	9, 10, 11, 12, 13, 30, 31, 32, 33, 34, 35, 38, 41B, 41M, 43O, 43I, 49O, 49I, 50, 51O, 51I, 52, 57, 58, 61
Concentricity	X	6M, 6T
