# Supplementary material for: Tri-Planar Geometric Dimensioning and Tolerancing Characteristics of SS 316L Laser Powder Bed Fusion Process Test Artifacts and Effect of Base Plate Removal
Source: Materials (Basel). 2021 Jun 26;14(13):3575. doi: 10.3390/ma14133575 (PMC8269720; doi:10.3390/ma14133575)
Supplement: Supplementary file 1 [file materials-14-03575-s001.zip › materials-1223197-supplementary.pdf]

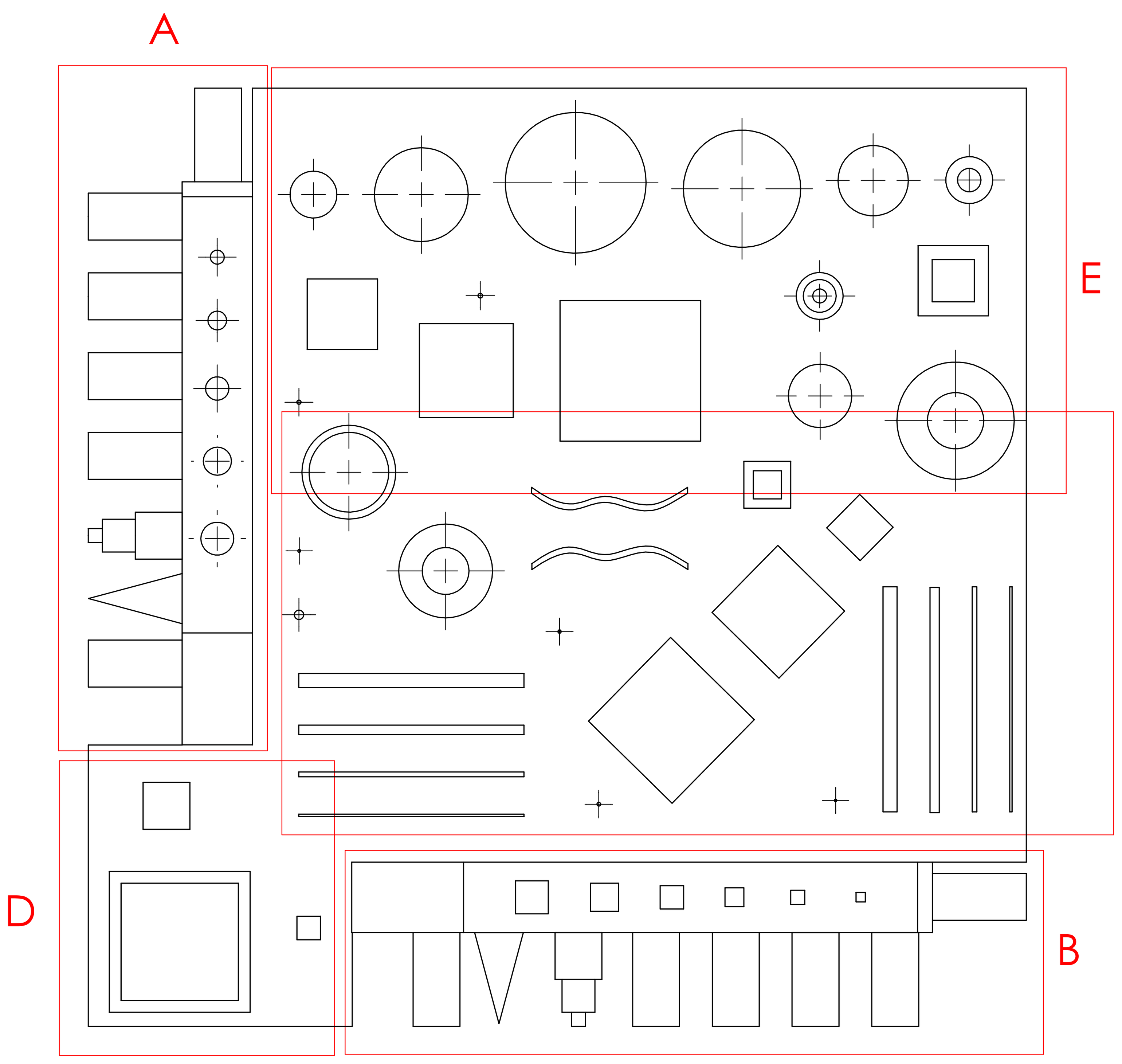

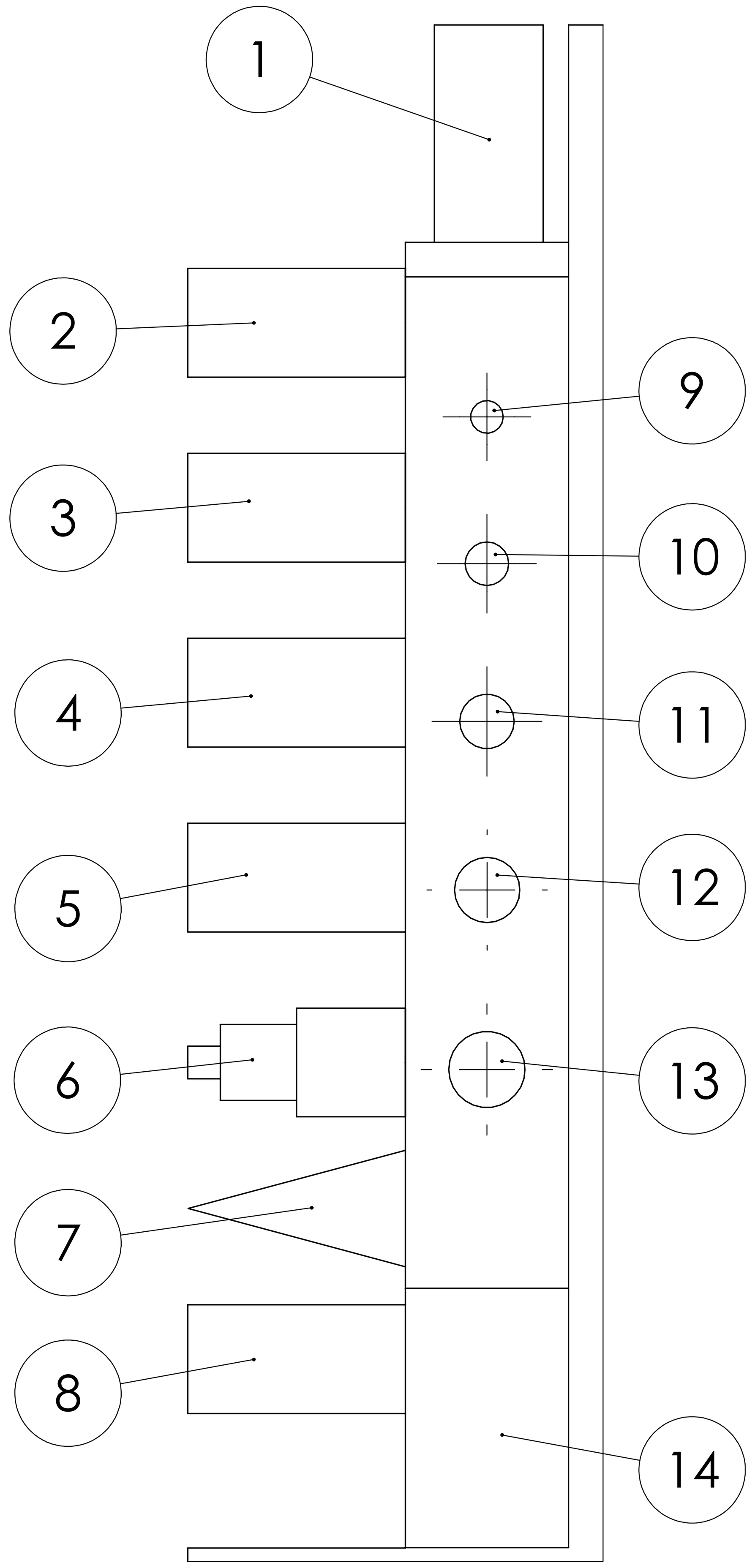

DETAIL A
SCALE 2 : 1

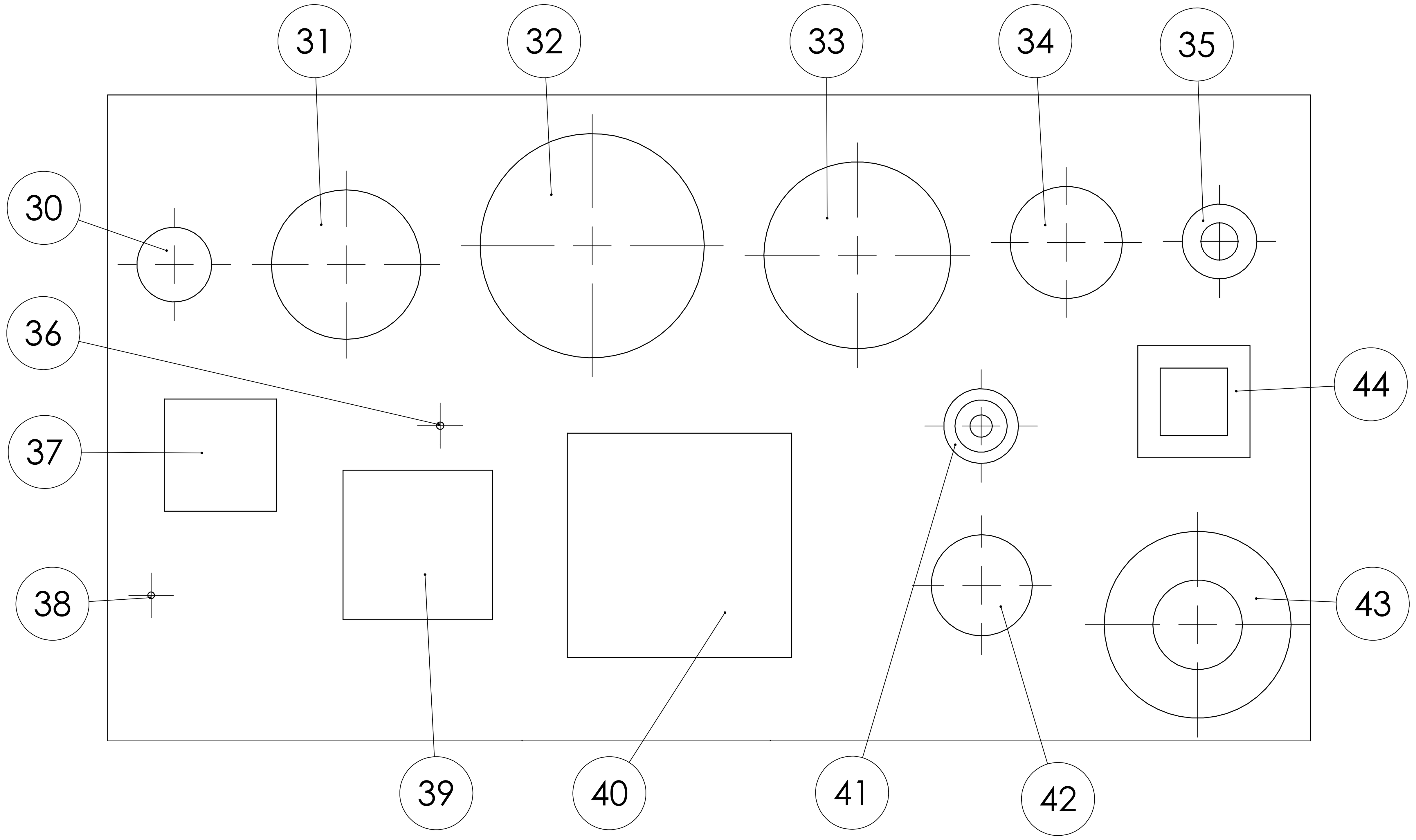

DETAIL E
SCALE 2 : 1

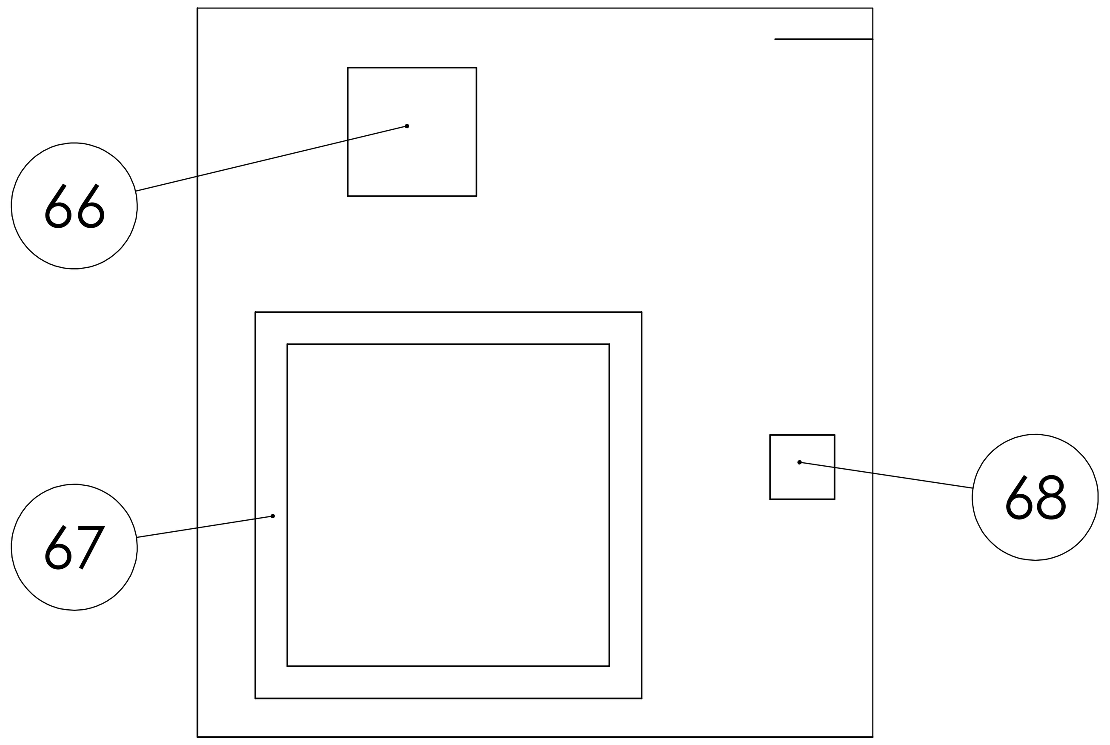

DETAIL D
SCALE 2 : 1

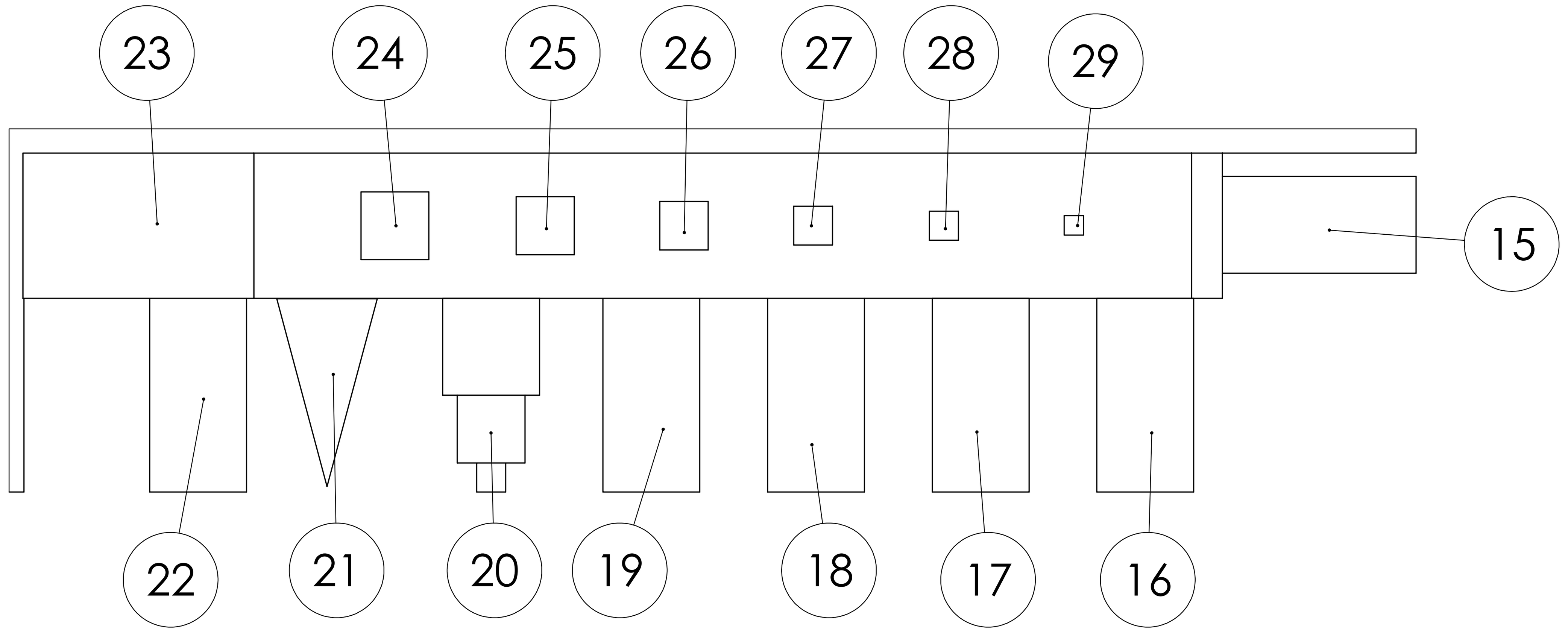

DETAIL B
SCALE 2 : 1

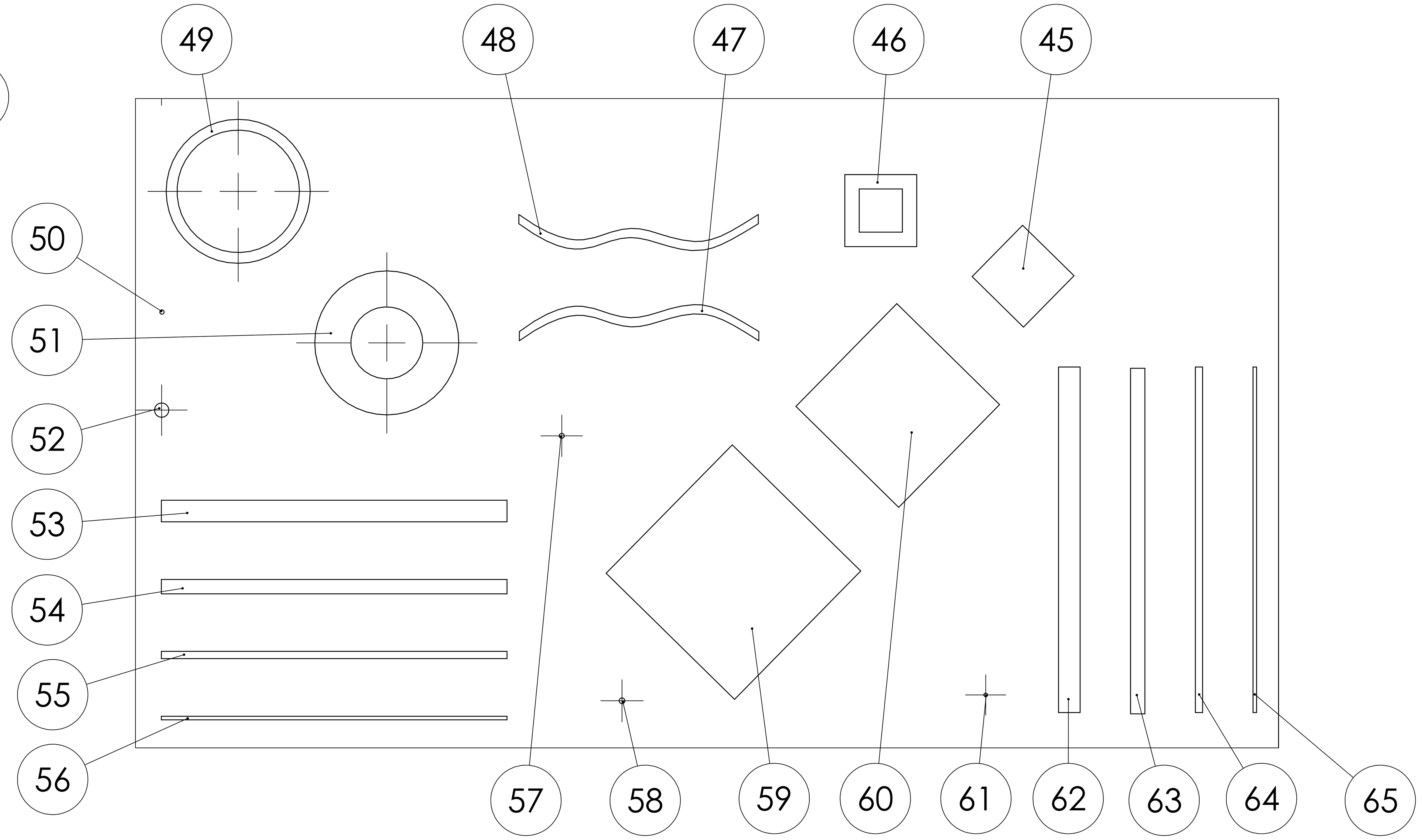

DETAIL C
SCALE 2 : 1

SOLIDWORKS Educational Product. For Instructional Use Only.

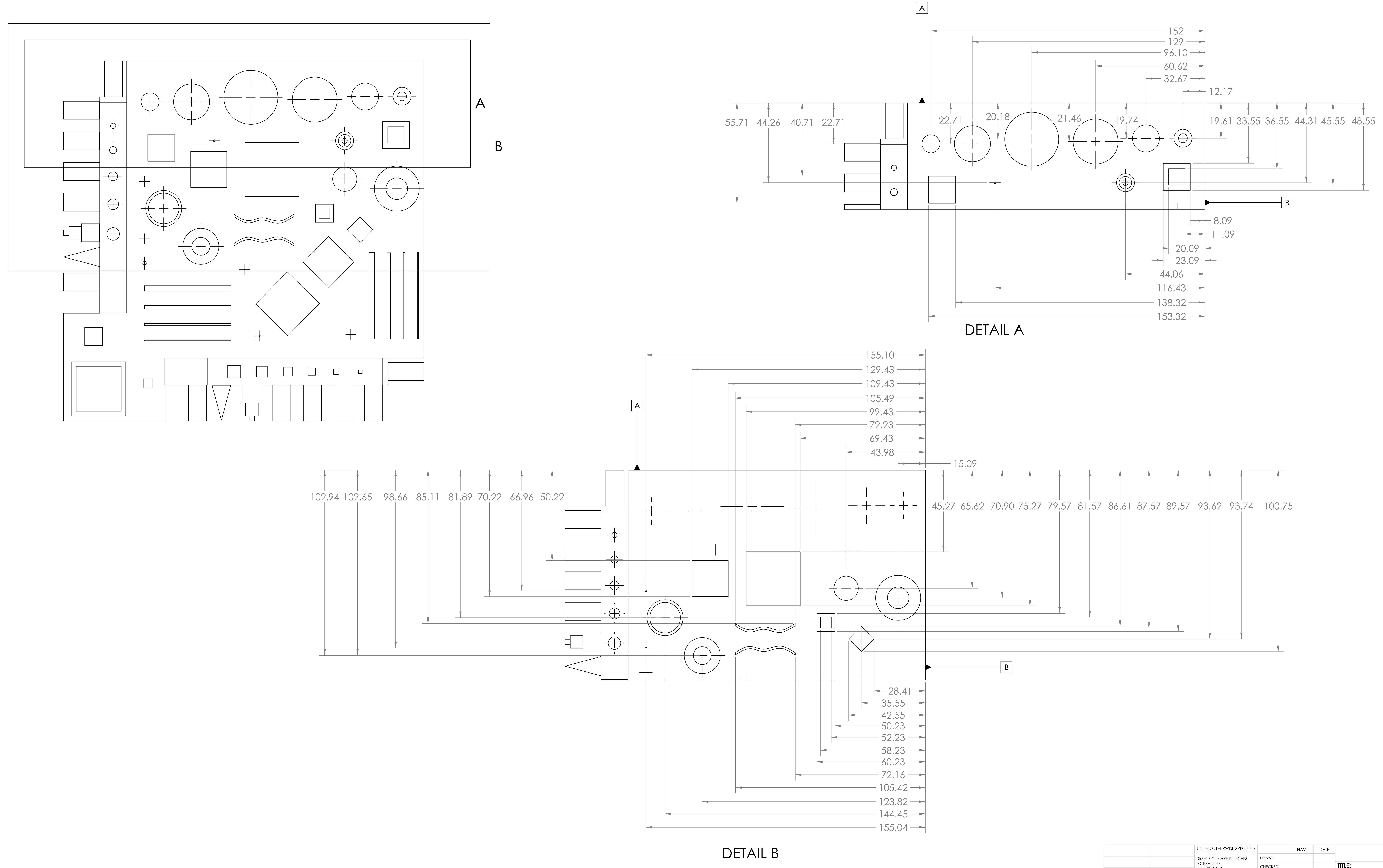

PROPRIETARY AND CONFIDENTIAL

THE INFORMATION CONTAINED IN THIS DRAWING IS THE SOLE PROPERTY OF <INSERT COMPANY NAME HERE>. ANY REPRODUCTION IN PART OR AS A WHOLE WITHOUT THE WRITTEN PERMISSION OF <INSERT COMPANY NAME HERE> IS PROHIBITED.

NEXT ASSY	USED ON	UNLESS OTHERWISE SPECIFIED: DIMENSIONS ARE IN INCHES TOLERANCES: FRACTIONAL ± ANGULAR: MACH ± BEND ± TWO PLACE DECIMAL ± THREE PLACE DECIMAL ±		NAME	DATE
APPLICATION		INTERPRET GEOMETRIC TOLERANCING PER:	DRAWN		
		MATERIAL Material <not specified>	CHECKED		
		FINISH	ENG APPR.		
		DO NOT SCALE DRAWING	MFG APPR.		
			Q.A.		
			COMMENTS:		

TITLE: Geometric Optimization of Metal 3D Printed Assemblies

SIZE	DWG. NO.	REV
D	1	
SCALE: 1:1	WEIGHT:	SHEET 1 OF 1

SOLIDWORKS Educational Product. For Instructional Use Only.

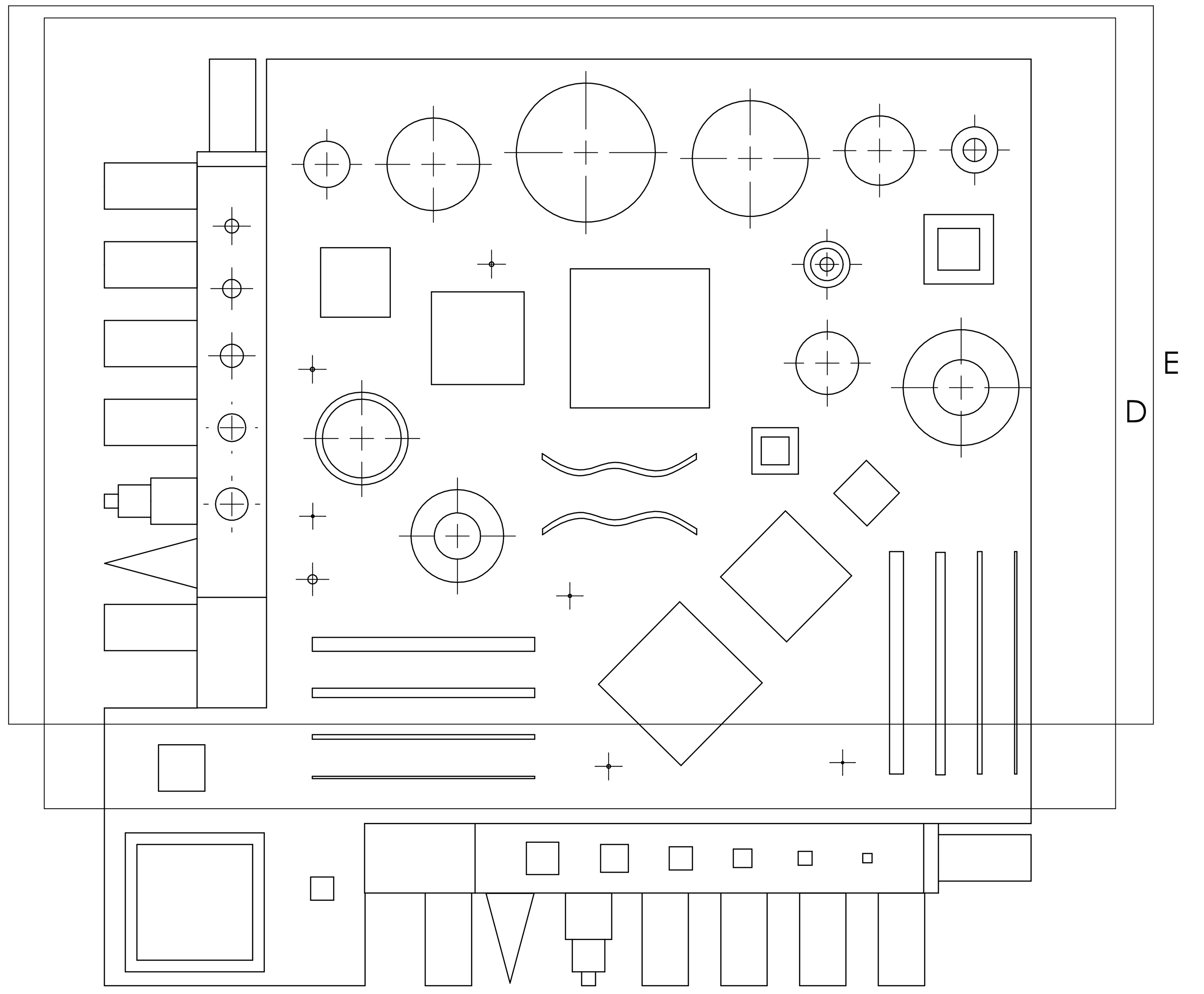

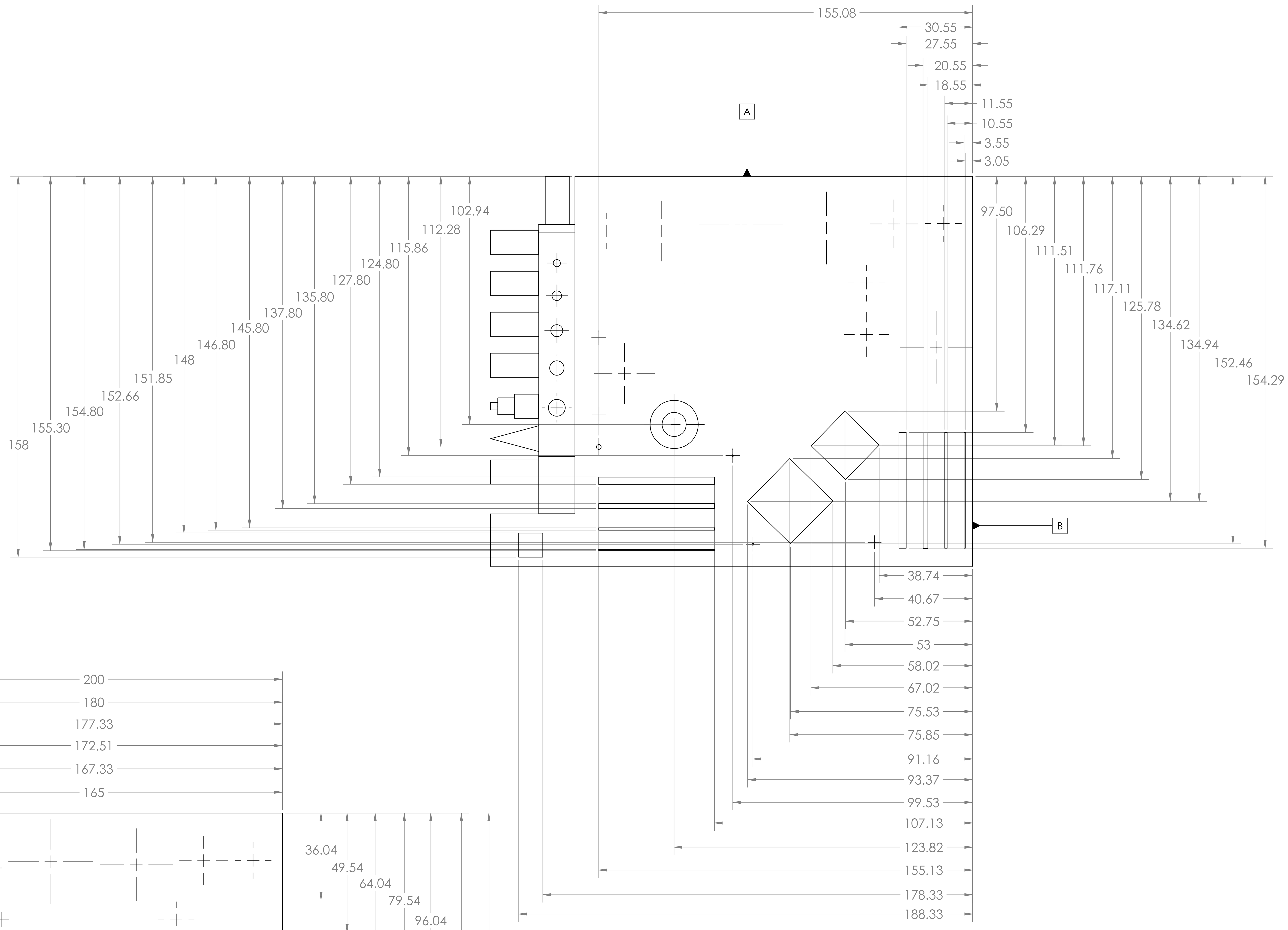

DETAIL D

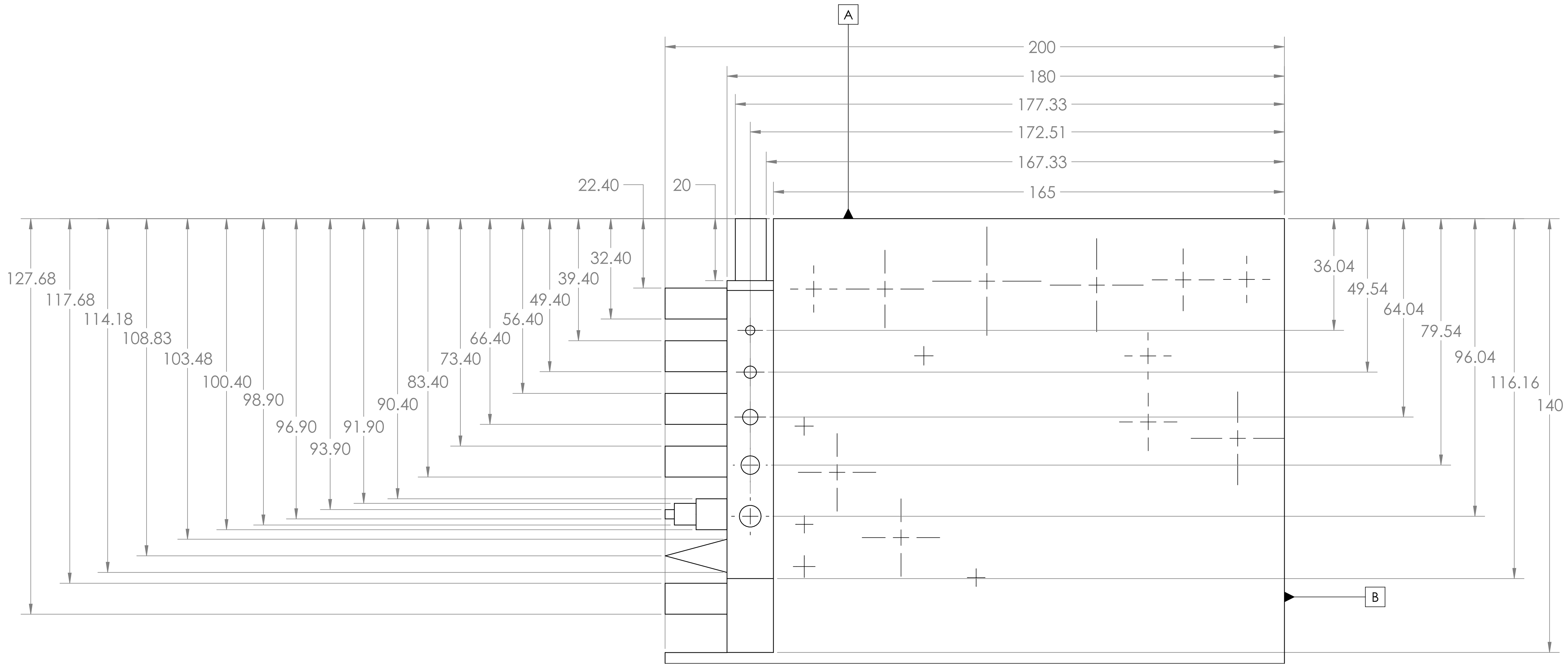

DETAIL E

PROPRIETARY AND CONFIDENTIAL
THE INFORMATION CONTAINED IN THIS DRAWING IS THE SOLE PROPERTY OF <INSERT COMPANY NAME HERE>. ANY REPRODUCTION IN PART OR AS A WHOLE WITHOUT THE WRITTEN PERMISSION OF <INSERT COMPANY NAME HERE> IS PROHIBITED.

NEXT ASSY	USED ON	
APPLICATION		

UNLESS OTHERWISE SPECIFIED:
DIMENSIONS ARE IN INCHES
TOLERANCES:
FRACTIONAL ±
ANGULAR: MACH ± BEND ±
TWO PLACE DECIMAL ±
THREE PLACE DECIMAL ±
INTERPRET GEOMETRIC TOLERANCING PER:
MATERIAL
Material <not specified>
FINISH
DO NOT SCALE DRAWING

	NAME	DATE
DRAWN		
CHECKED		
ENG APPR.		
MFG APPR.		
Q.A.		
COMMENTS:		

TITLE:
Geometric Optimization of Metal 3D Printed Assemblies

SIZE D | DWG. NO. 2 | REV

SCALE: 1:1 | WEIGHT: | SHEET 1 OF 1

SOLIDWORKS Educational Product. For Instructional Use Only.

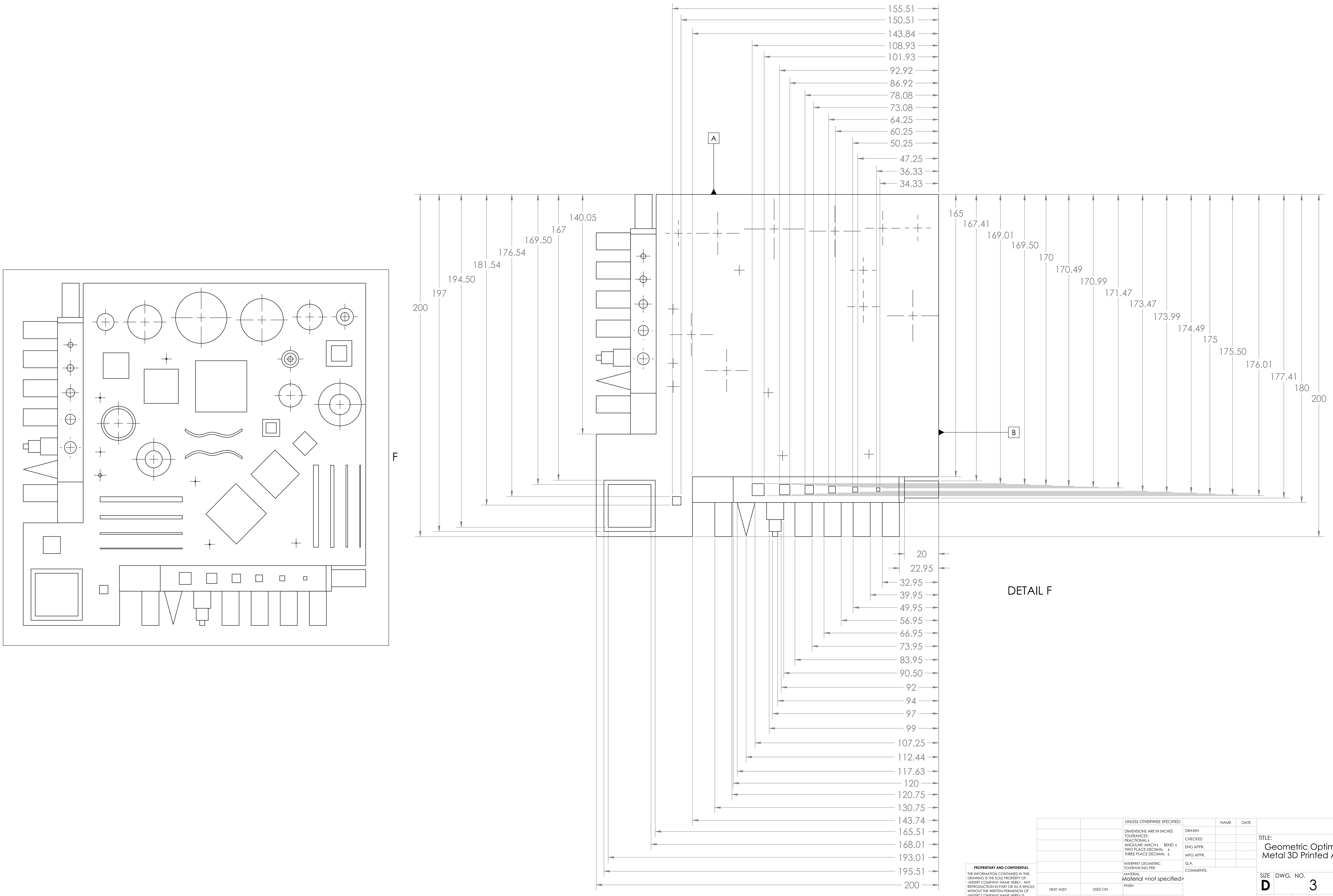

UNLESS OTHERWISE SPECIFIED:		NAME	DATE
DIMENSIONS ARE IN INCHES<br>TOLERANCES:<br>FRACTIONAL ±<br>ANGULAR: MACH ± BEND ±<br>TWO PLACE DECIMAL ±<br>THREE PLACE DECIMAL ±	DRAWN		
	CHECKED		
	ENG APPR.		
	MFG APPR.		
INTERPRET GEOMETRIC TOLERANCING PER:	Q.A.		
MATERIAL<br>Material <not specified>	COMMENTS:		
FINISH			
DO NOT SCALE DRAWING			

NEXT ASSY	USED ON
APPLICATION	

PROPRIETARY AND CONFIDENTIAL

THE INFORMATION CONTAINED IN THIS DRAWING IS THE SOLE PROPERTY OF <INSERT COMPANY NAME HERE>. ANY REPRODUCTION IN PART OR AS A WHOLE WITHOUT THE WRITTEN PERMISSION OF <INSERT COMPANY NAME HERE> IS PROHIBITED.

TITLE: Geometric Optimization of Metal 3D Printed Assemblies

SIZE: D | DWG. NO. 3 | REV

SCALE: 1:1 | WEIGHT: | SHEET 1 OF 1

SOLIDWORKS Educational Product. For Instructional Use Only.

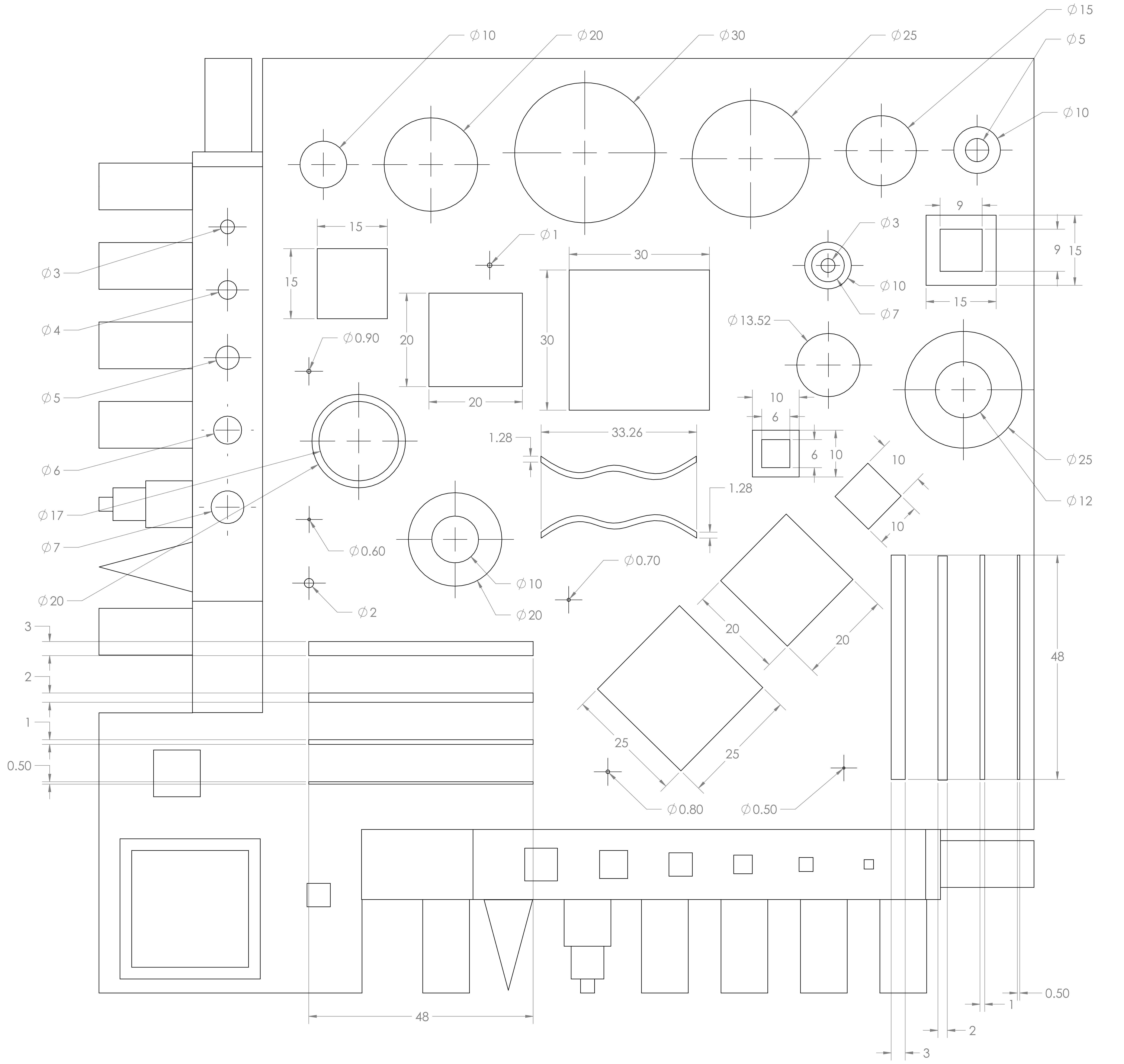

		UNLESS OTHERWISE SPECIFIED:		NAME	DATE
		DIMENSIONS ARE IN INCHES<br>TOLERANCES:<br>FRACTIONAL ±<br>ANGULAR: MACH ± BEND ±<br>TWO PLACE DECIMAL ±<br>THREE PLACE DECIMAL ±	DRAWN		
			CHECKED		
			ENG APPR.		
			MFG APPR.		
		INTERPRET GEOMETRIC TOLERANCING PER:	Q.A.		
		MATERIAL<br>Material <not specified>	COMMENTS:		
NEXT ASSY	USED ON	FINISH			
APPLICATION		DO NOT SCALE DRAWING			

PROPRIETARY AND CONFIDENTIAL

THE INFORMATION CONTAINED IN THIS DRAWING IS THE SOLE PROPERTY OF <INSERT COMPANY NAME HERE>. ANY REPRODUCTION IN PART OR AS A WHOLE WITHOUT THE WRITTEN PERMISSION OF <INSERT COMPANY NAME HERE> IS PROHIBITED.

TITLE:
Geometric Optimization of Metal 3D Printed Assemblies

SIZE	DWG. NO.	REV
D	4	
SCALE: 1:1	WEIGHT:	SHEET 1 OF 1

SOLIDWORKS Educational Product. For Instructional Use Only.

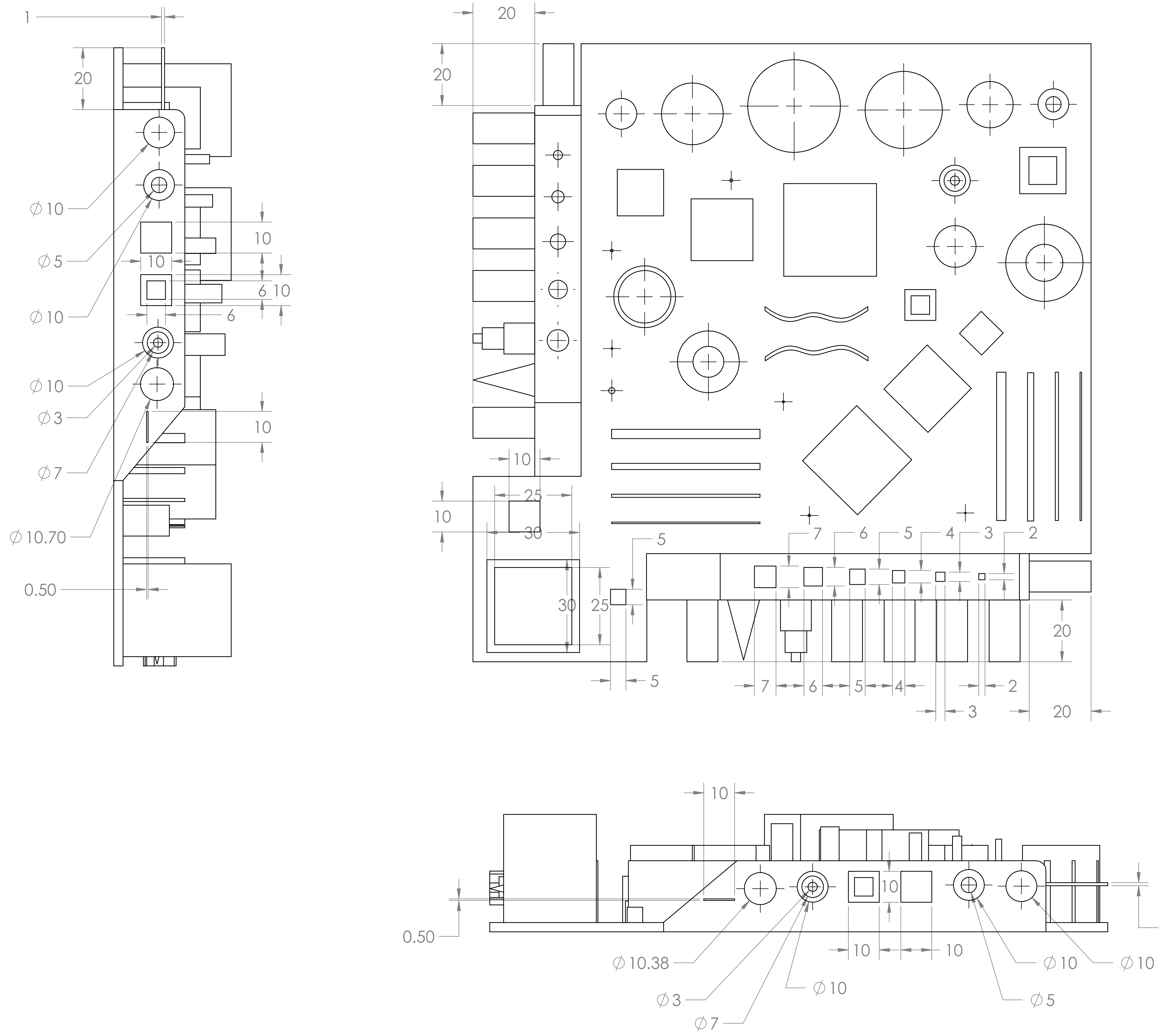

UNLESS OTHERWISE SPECIFIED:		NAME	DATE
DIMENSIONS ARE IN INCHES TOLERANCES: FRACTIONAL± ANGULAR: MACH± BEND ± TWO PLACE DECIMAL ± THREE PLACE DECIMAL ±	DRAWN		
	CHECKED		
	ENG APPR.		
	MFG APPR.		
INTERPRET GEOMETRIC TOLERANCING PER:	Q.A.		
MATERIAL Material <not specified>	COMMENTS:		
FINISH			
DO NOT SCALE DRAWING			

NEXT ASSY | USED ON

APPLICATION

PROPRIETARY AND CONFIDENTIAL

THE INFORMATION CONTAINED IN THIS DRAWING IS THE SOLE PROPERTY OF <INSERT COMPANY NAME HERE>. ANY REPRODUCTION IN PART OR AS A WHOLE WITHOUT THE WRITTEN PERMISSION OF <INSERT COMPANY NAME HERE> IS PROHIBITED.

TITLE:

Geometric Optimization of Metal 3D Printed Assemblies

SIZE	DWG. NO.	REV
C	5	
SCALE: 1:1	WEIGHT:	SHEET 1 OF 1

SOLIDWORKS Educational Product. For Instructional Use Only.

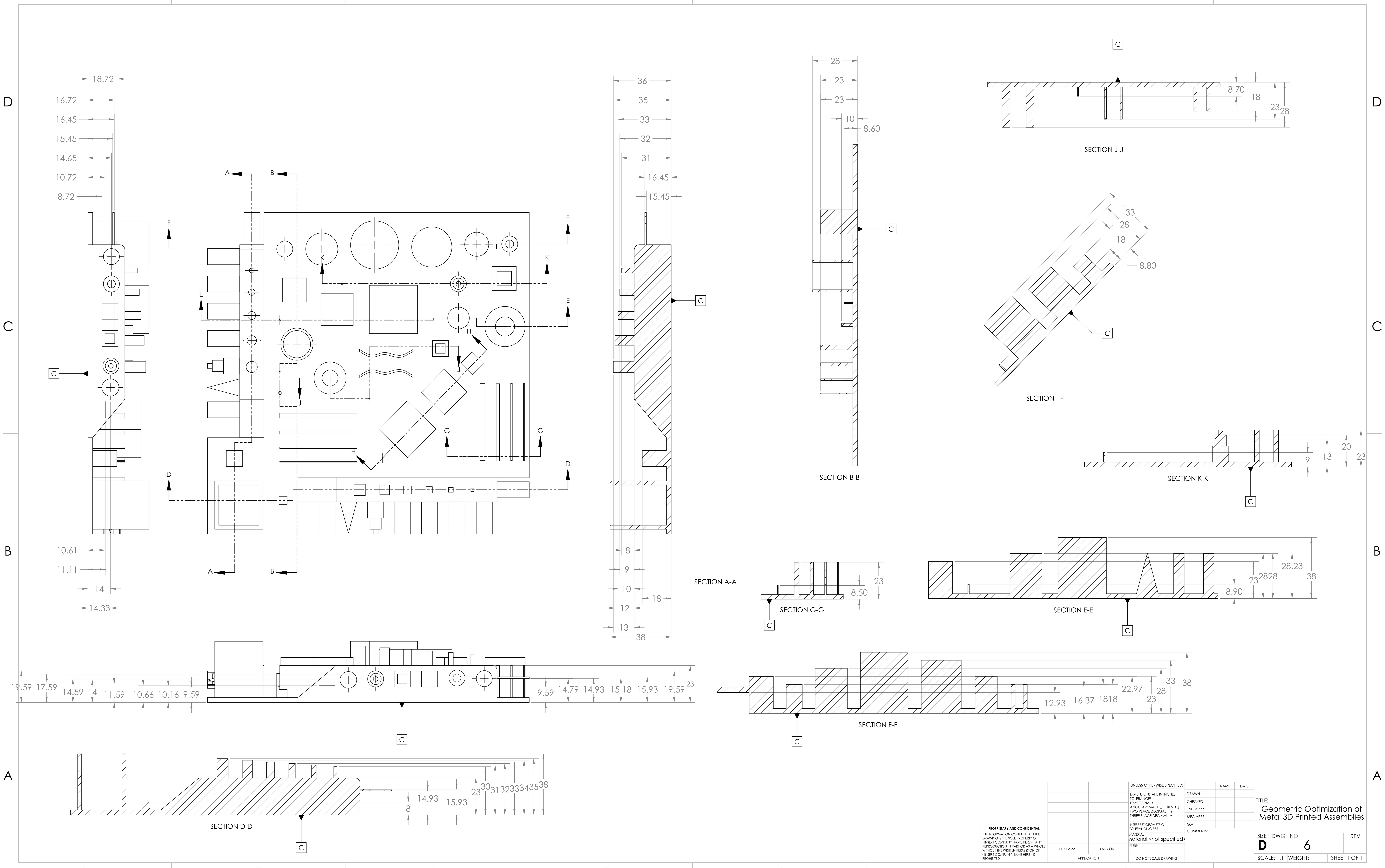

SOLIDWORKS Educational Product. For Instructional Use Only.

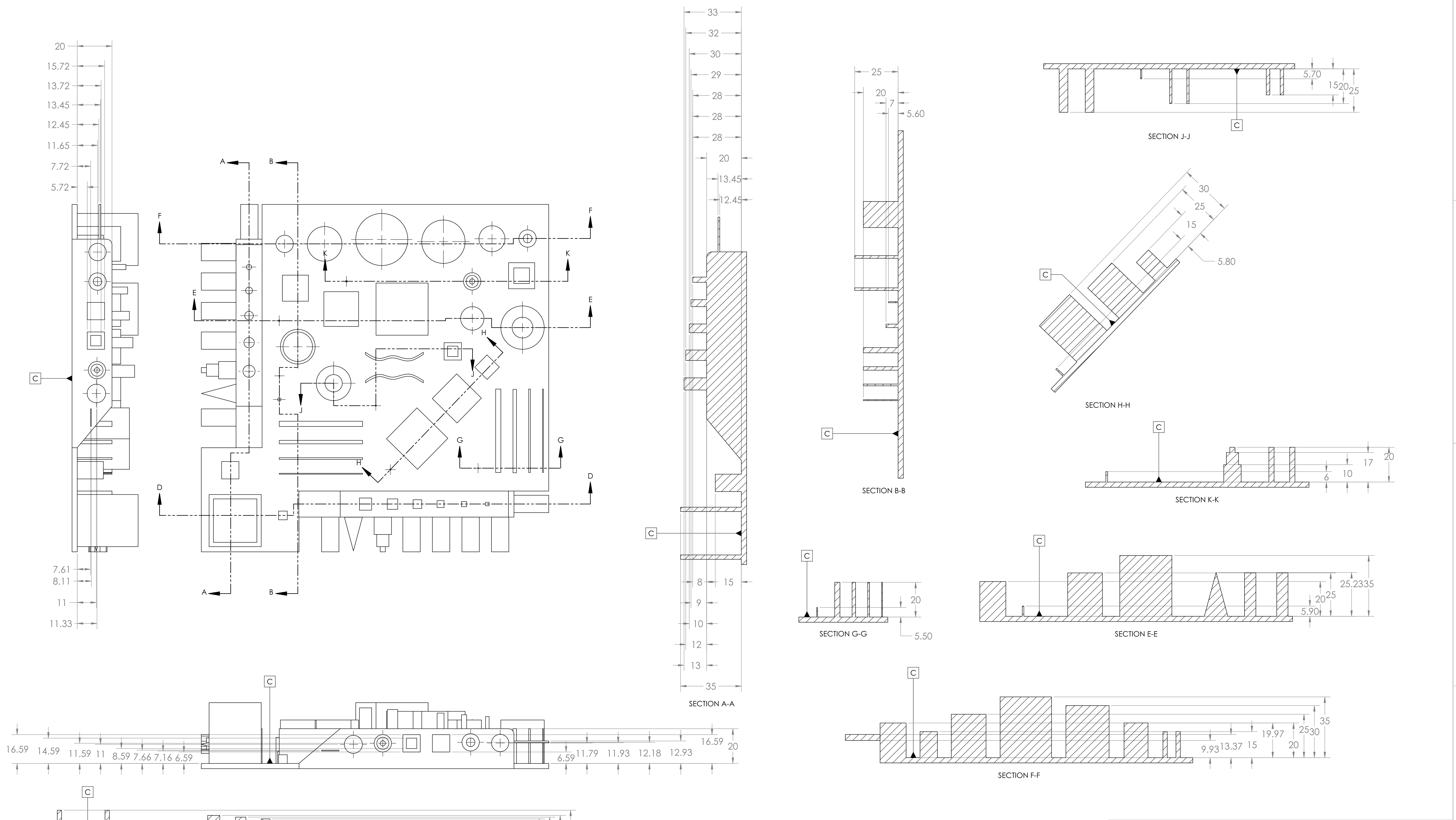

PROPRIETARY AND CONFIDENTIAL

THE INFORMATION CONTAINED IN THIS DRAWING IS THE SOLE PROPERTY OF <INSERT COMPANY NAME HERE>. ANY REPRODUCTION IN PART OR AS A WHOLE WITHOUT THE WRITTEN PERMISSION OF <INSERT COMPANY NAME HERE> IS PROHIBITED.

NEXT ASSY	USED ON
APPLICATION	

UNLESS OTHERWISE SPECIFIED:
DIMENSIONS ARE IN INCHES
TOLERANCES:
FRACTIONAL ±
ANGULAR: MACH ± BEND ±
TWO PLACE DECIMAL ±
THREE PLACE DECIMAL ±

INTERPRET GEOMETRIC TOLERANCING PER:

MATERIAL
Material <not specified>

FINISH

DO NOT SCALE DRAWING

	NAME	DATE
DRAWN		
CHECKED		
ENG APPR.		
MFG APPR.		
Q.A.		
COMMENTS:		

TITLE:
Geometric Optimization of Metal 3D Printed Assemblies

SIZE	DWG. NO.	REV
D	7	

SCALE: 1:1 WEIGHT: SHEET 1 OF 1

SOLIDWORKS Educational Product. For Instructional Use Only.

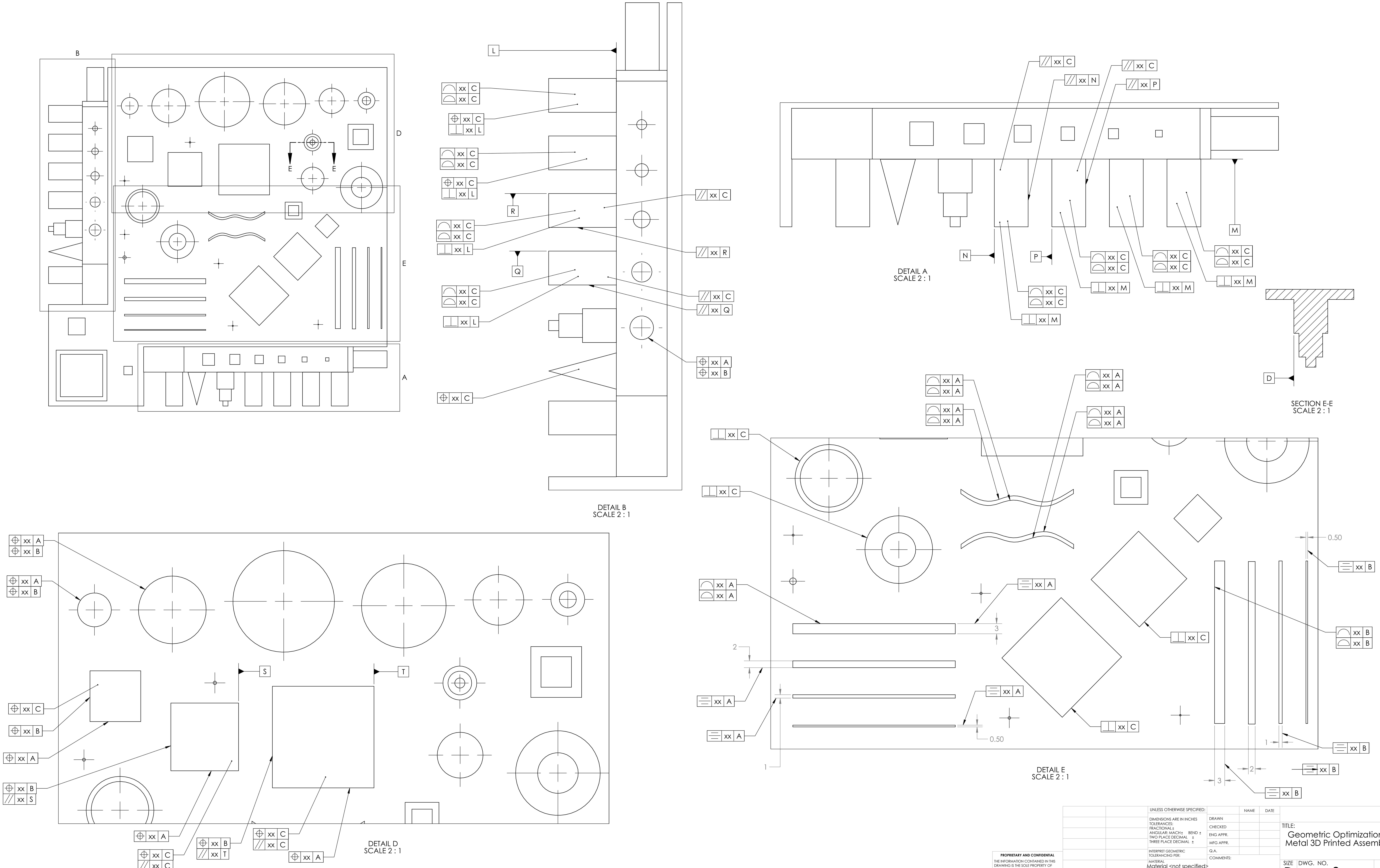

PROPRIETARY AND CONFIDENTIAL

THE INFORMATION CONTAINED IN THIS DRAWING IS THE SOLE PROPERTY OF <INSERT COMPANY NAME HERE>. ANY REPRODUCTION IN PART OR AS A WHOLE WITHOUT THE WRITTEN PERMISSION OF <INSERT COMPANY NAME HERE> IS PROHIBITED.

		UNLESS OTHERWISE SPECIFIED:		NAME	DATE	
		DIMENSIONS ARE IN INCHES<br>TOLERANCES:<br>FRACTIONAL ±<br>ANGULAR: MACH ± BEND ±<br>TWO PLACE DECIMAL ±<br>THREE PLACE DECIMAL ±	DRAWN			
			CHECKED			TITLE: Geometric Optimization of Metal 3D Printed Assemblies
			ENG APPR.			
			MFG APPR.			
		INTERPRET GEOMETRIC TOLERANCING PER:	Q.A.			
		MATERIAL<br>Material <not specified>	COMMENTS:			SIZE D / DWG. NO. 8 / REV
NEXT ASSY	USED ON	FINISH				
APPLICATION		DO NOT SCALE DRAWING				SCALE: 1:1 / WEIGHT: / SHEET 1 OF 1

SOLIDWORKS Educational Product. For Instructional Use Only.

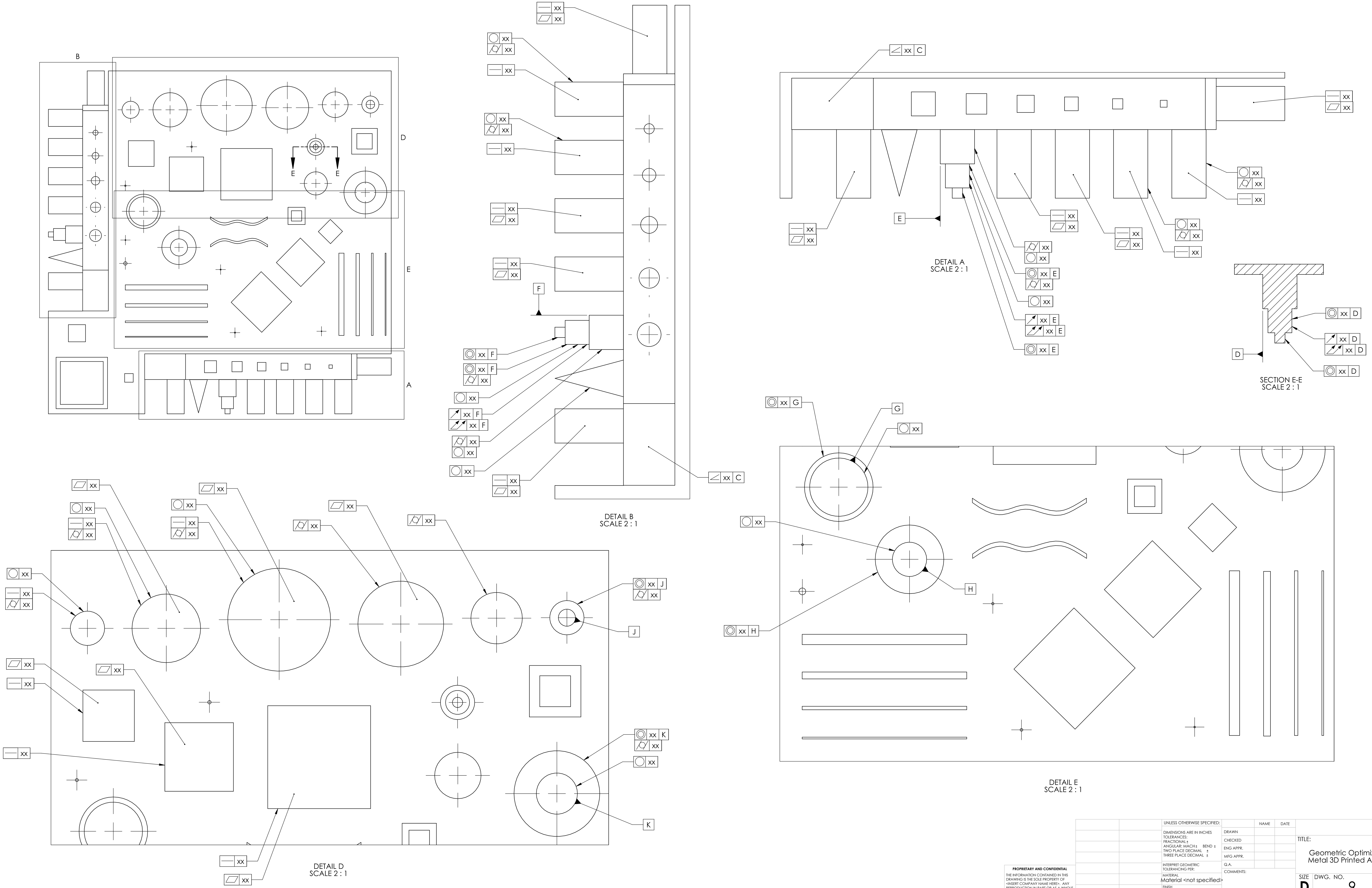

PROPRIETARY AND CONFIDENTIAL

THE INFORMATION CONTAINED IN THIS DRAWING IS THE SOLE PROPERTY OF <INSERT COMPANY NAME HERE>. ANY REPRODUCTION IN PART OR AS A WHOLE WITHOUT THE WRITTEN PERMISSION OF <INSERT COMPANY NAME HERE> IS PROHIBITED.

NEXT ASSY	USED ON
APPLICATION	

UNLESS OTHERWISE SPECIFIED:
DIMENSIONS ARE IN INCHES
TOLERANCES:
FRACTIONAL ±
ANGULAR: MACH ± BEND ±
TWO PLACE DECIMAL ±
THREE PLACE DECIMAL ±

INTERPRET GEOMETRIC TOLERANCING PER:

MATERIAL
Material <not specified>

FINISH

DO NOT SCALE DRAWING

	NAME	DATE
DRAWN		
CHECKED		
ENG APPR.		
MFG APPR.		
Q.A.		

COMMENTS:

TITLE:
Geometric Optimization of Metal 3D Printed Assemblies

SIZE	DWG. NO.	REV
D	9	

SCALE: 1:1 WEIGHT: SHEET 1 OF 1

SOLIDWORKS Educational Product. For Instructional Use Only.

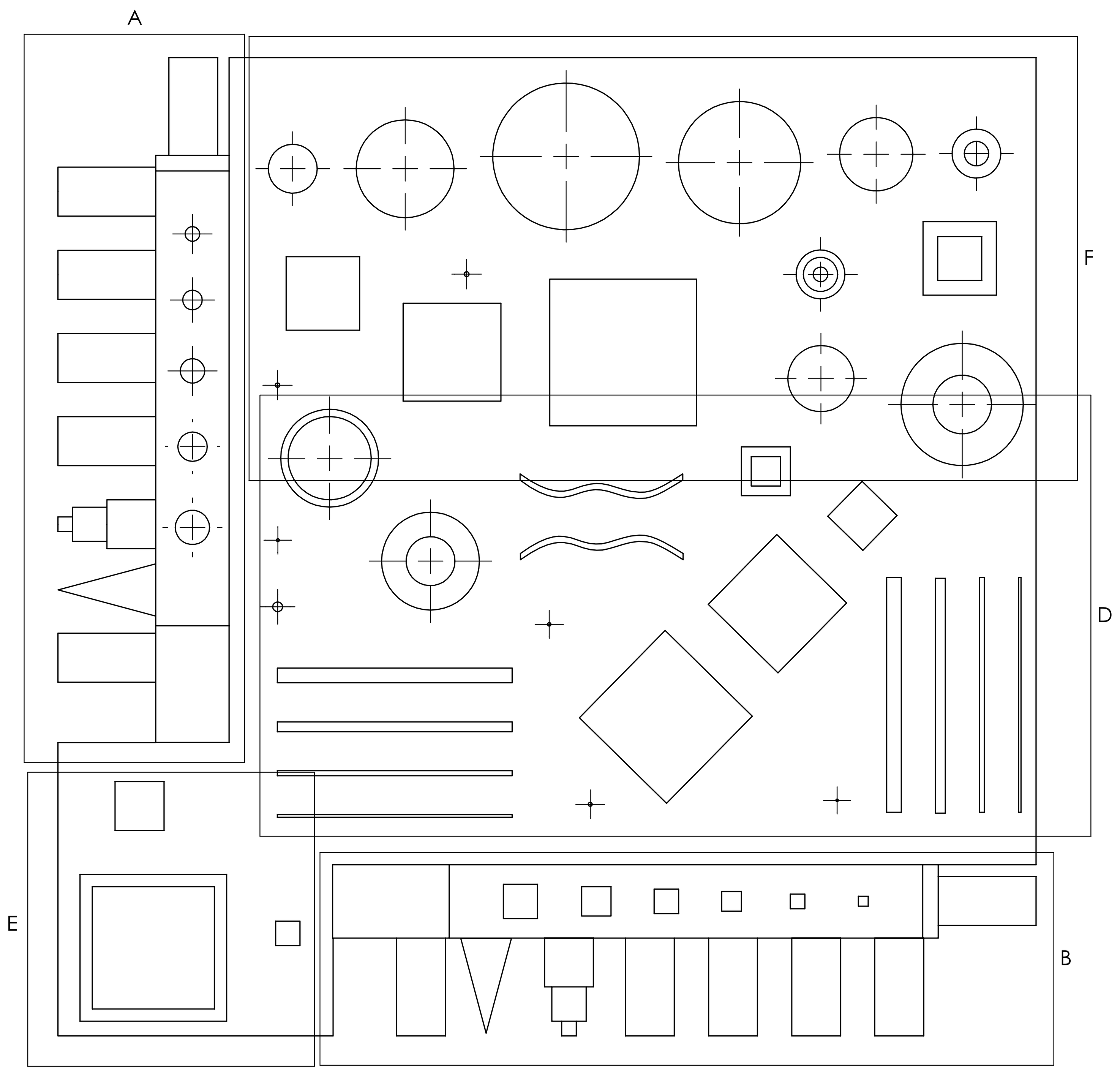

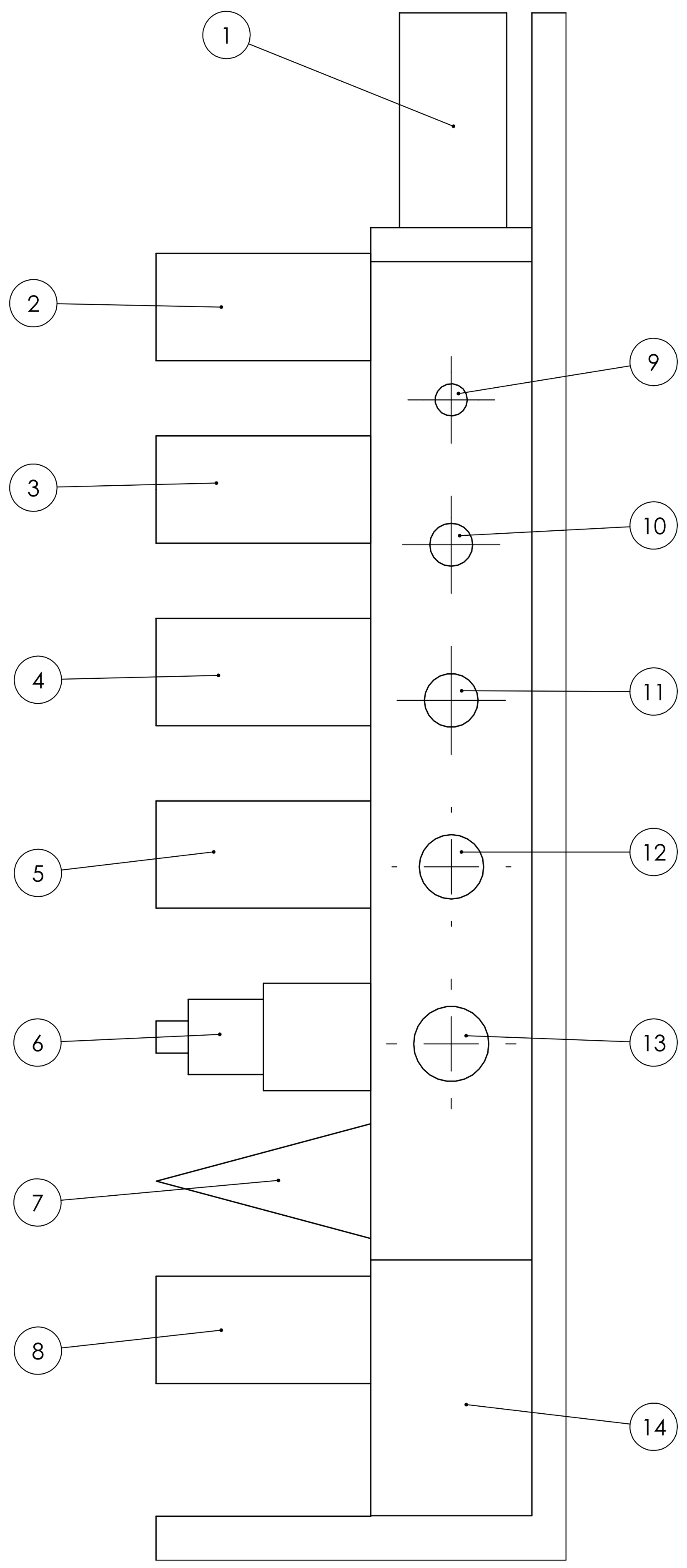

DETAIL A
SCALE 2 : 1

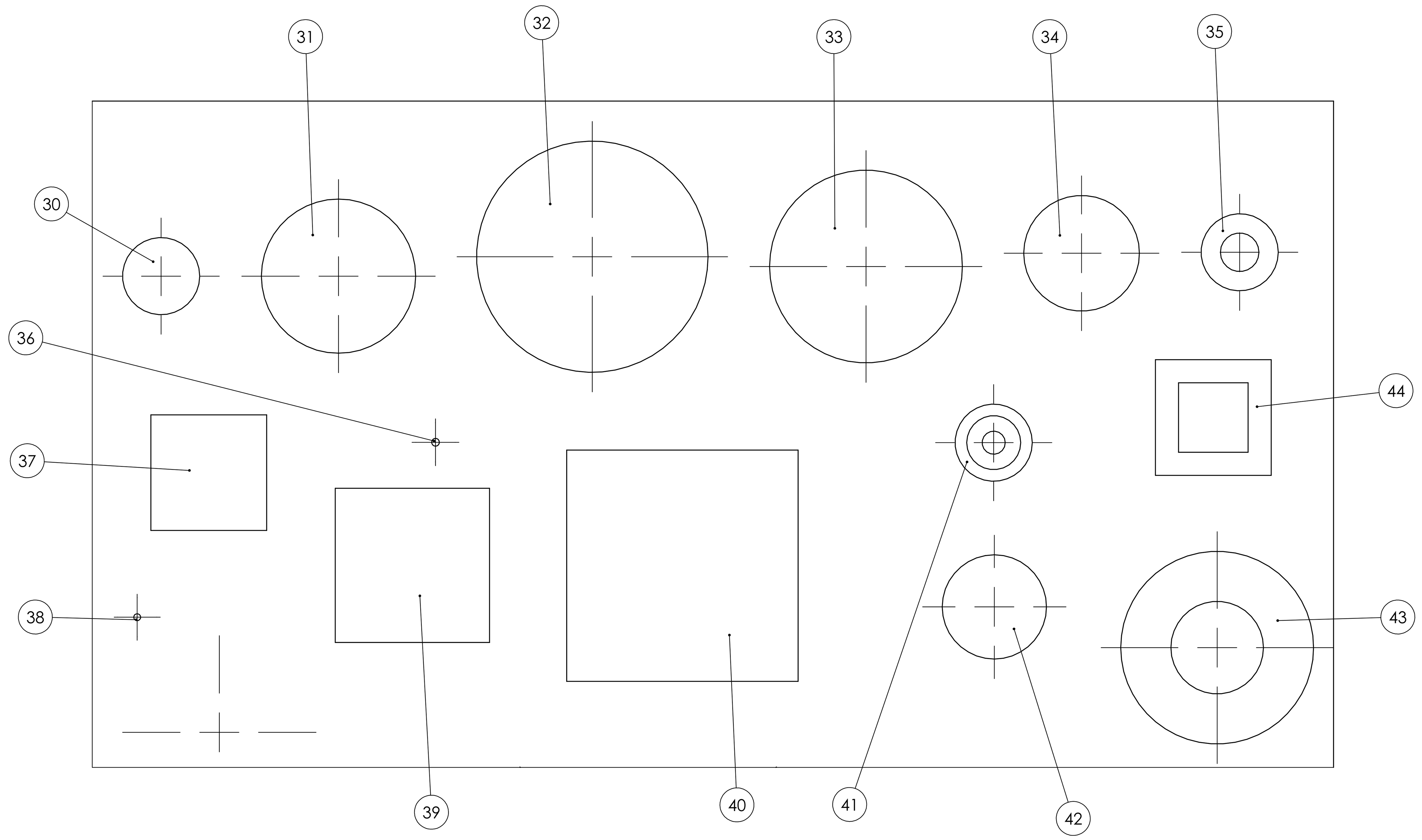

DETAIL F
SCALE 2 : 1

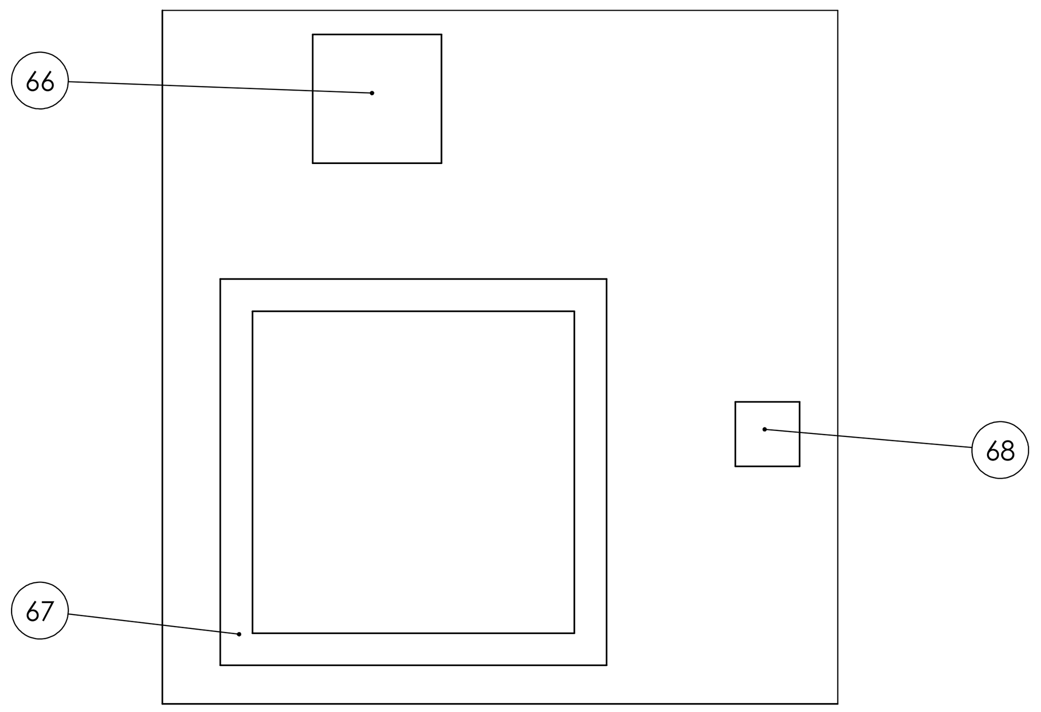

DETAIL E
SCALE 2 : 1

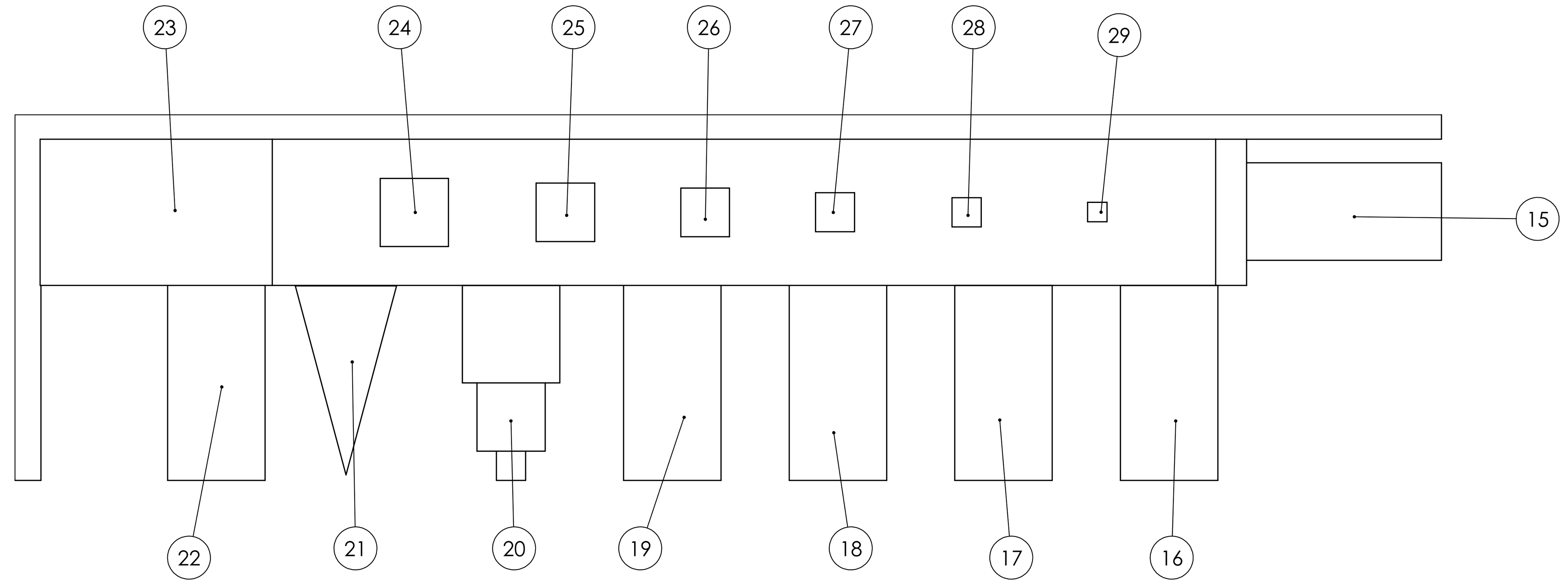

DETAIL B
SCALE 2 : 1

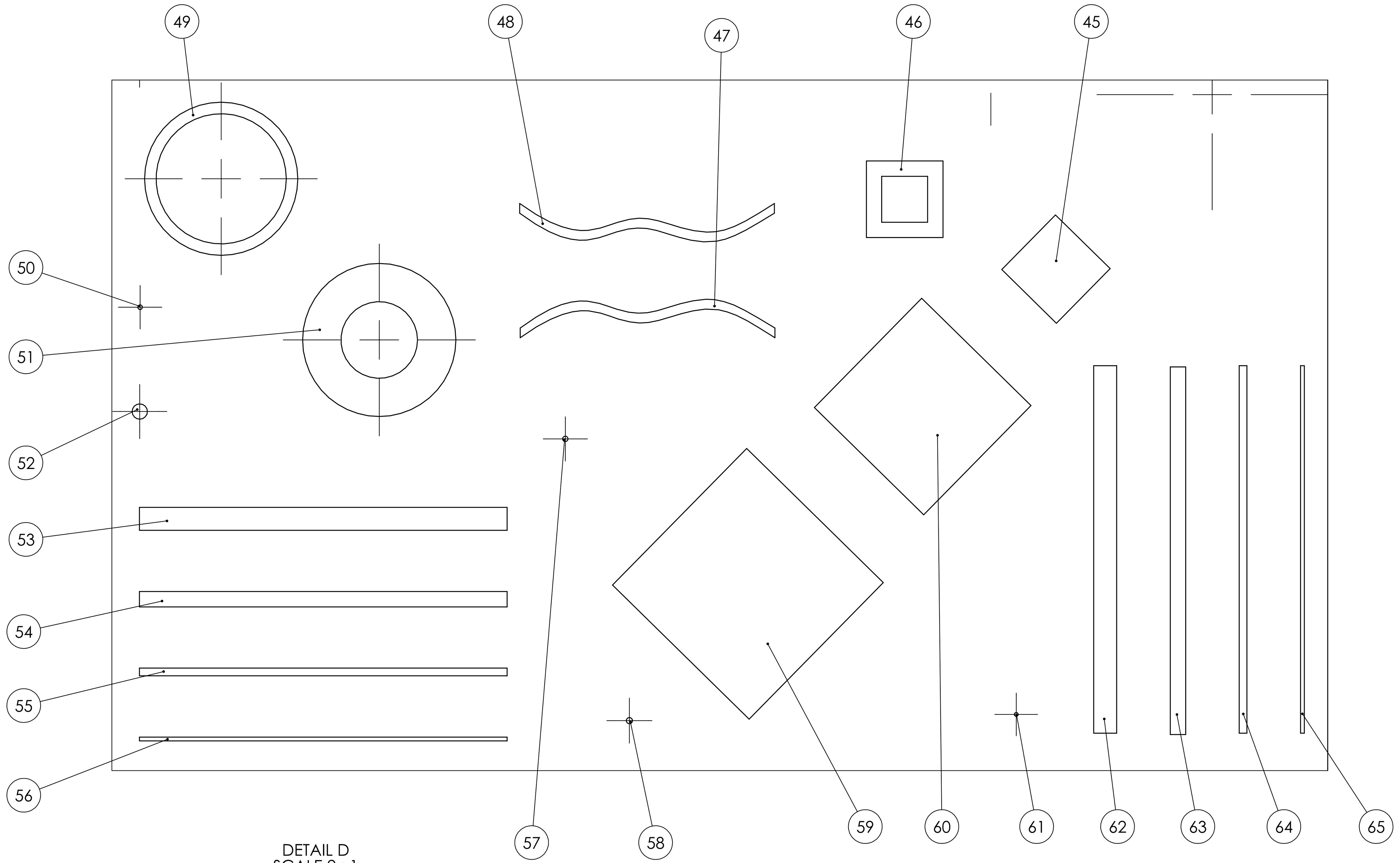

DETAIL D
SCALE 2 : 1

PROPRIETARY AND CONFIDENTIAL
THE INFORMATION CONTAINED IN THIS DRAWING IS THE SOLE PROPERTY OF <INSERT COMPANY NAME HERE>. ANY REPRODUCTION IN PART OR AS A WHOLE WITHOUT THE WRITTEN PERMISSION OF <INSERT COMPANY NAME HERE> IS PROHIBITED.

UNLESS OTHERWISE SPECIFIED:
DIMENSIONS ARE IN INCHES
TOLERANCES:
FRACTIONAL ±
ANGULAR: MACH ± BEND ±
TWO PLACE DECIMAL ±
THREE PLACE DECIMAL ±
INTERPRET GEOMETRIC TOLERANCING PER:
MATERIAL
Material <not specified>
FINISH
NEXT ASSY | USED ON
APPLICATION
DO NOT SCALE DRAWING

	NAME	DATE
DRAWN		
CHECKED		
ENG APPR.		
MFG APPR.		
Q.A.		
COMMENTS:		

TITLE:
Geometric Optimization of Metal 3D Printed Assemblies

SIZE D | DWG. NO. 10 | REV
SCALE: 1:1 | WEIGHT: | SHEET 1 OF 1

SOLIDWORKS Educational Product. For Instructional Use Only.
